# Analytical study of reaction diffusion Lengyel-Epstein system by generalized Riccati equation mapping method

**DOI:** 10.1038/s41598-023-47207-4

**Published:** 2023-11-16

**Authors:** Nauman Ahmed, Muhammad Z. Baber, Muhammad Sajid Iqbal, Amina Annum, Syed Mansoor Ali, Mubasher Ali, Ali Akgül, Sayed M. El Din

**Affiliations:** 1 Department of Mathematics and Statistics, The University of Lahore, Lahore, Pakistan; 2 Department of Computer Science and Mathematics, Lebanese American University, Beirut, Lebanon; 3 Mathematics Research Center, Department of Mathematics, Near East University, Near East Boulevard, PC: 99138 Nicosia/Mersin 10, Turkey; 4 Department of Humanities and Basic Science, MCS, National University of Science and Technology, Islamabad, Pakistan; 5 Department of Academic Affairs, School of Leadership and Business, Oryx Universal College With Liverpool John Moores University (UK), Doha, 12253 Qatar; 6 Department of Physics and Astronomy, College of Science, King Saud University, P.O. BOX 2455, 11451 Riyadh, Saudi Arabia; 7 Scool of Engineering and Digital Arts, University of Kent, Canterbury Kent, UK; 8 Art and Science Faculty, Department of Mathematics, Siirt University, TR-56100 Siirt, Turkey; 9 Center of Research, Faculty of Engineering, Future University in Egypt, New Catiro, 11835 Egypt

**Keywords:** Mathematics and computing, Functional clustering

## Abstract

In this study, the Lengyel-Epstein system is under investigation analytically. This is the reaction–diffusion system leading to the concentration of the inhibitor chlorite and the activator iodide, respectively. These concentrations of the inhibitor chlorite and the activator iodide are shown in the form of wave solutions. This is a reactionâ€ “diffusion model which considered for the first time analytically to explore the different abundant families of solitary wave structures. These exact solitary wave solutions are obtained by applying the generalized Riccati equation mapping method. The single and combined wave solutions are observed in shock, complex solitary-shock, shock singular, and periodic-singular forms. The rational solutions also emerged during the derivation. In the Lengyel-Epstein system, solitary waves can propagate at various rates. The harmony of the system’s diffusive and reactive effects frequently governs the speed of a single wave. Solitary waves can move at a variety of speeds depending on the factors and reaction kinetics. To show their physical behavior, the 3D and their corresponding contour plots are drawn for the different values of constants.

## Introduction

Partial differential equations (PDEs) are widely used in sciences like mathematical physics, plasma physics, optics, quantum, mechanics, numerical analysis, and many engineering fields. The nonlinear PDEs describe the physical phenomena more accurately. Recently, it has become more important to determine the exact solutions, numerical and analytical solutions of non-linear PDEs with the help of using mathematical tools such as Maple, Mathematica, and MATLAB that ease solving complex and monotonous algebraic computations. Many mathematicians have developed methods to identify the exact solutions of nonlinear PDEs, including the Jacobi elliptic function method^1^, the generalized Riccati equation mapping method^2^, Rational Homotopy perturbation method^3^, and ${\phi }^{6}$-model expansion method^4,5^, the homogeneous balance method^6^, the tanh method^7^, the inverse scattering transform^8^, the exponential-function expansion method^9^, the Backlund transform^10^, the modified extended Fan subequation method^11^, the truncated Painleve expansion^12^, and the auxiliary equation method^13^. Ghanbari, B., et al., used the generalized exponential rational function method^14–22^, for the different nonlinear PDEs to investigated the different type of trigonometric, hyperbolic, exponential and rational solitary waves or soliton solutions. But in this study, the Generalized Riccati Equation Mapping (GREM) method is used. The GREM method is a powerful analytical technique for solving a wide range of differential equations, particularly nonlinear ones. It has several advantages, but it also comes with some limitations and potential disadvantages. the GREM method is a valuable analytical tool for solving a broad class of nonlinear differential equations. Its advantages include generality, nonlinearity handling, and the potential to reduce problems to simpler forms. However, its complexity, problem-specific nature, and computational demands can be disadvantages in certain situations. Researchers should consider their familiarity with GREM, the problem’s characteristics, and the desired level of analytical rigor when deciding whether to use this method. The list of actual applications of non-linear PDEs of extreme relevance and practical importance is long. One example that is plain sight is dynamic meteorology and numerical weather forecasting: the weather report you see every night on TV has been obtained from the numerical solution of a complex set of non-linear PDEs. Mathematical modeling is an essential part of simulation and design of control systems. Its main purpose is to be a simplified representation of real life, to mimic the applicable properties of the system being analyzed.

Reaction–diffusion systems are important in a wide range of disciplines. They arise naturally in chemistry and chemical engineering where they are used to represent the local mixing of different chemicals as well as the movement of substances through diffusion. However, they also have applications in the investigation of a variety of phenomena in the biological, ecological, environmental, life, and image processing sciences. There are many other types of reaction–diffusion systems in the literature, but Lengyel and Epstein’s model of the chlorite-iodide malonic acid (CIMA) reaction–diffusion has garnered a lot of interest lately. The revolutionary work by Turing in 1952^23^, which predicted the existence of stationary symmetry-breaking reaction–diffusion structures, also known as Turing patterns, is the foundation for the Lengyel-Epstein model. De Kepper’s work in 1990, however, is the first realization of Turing’s revolutionary work ever^24^. One of the most important studies on the Lengyel-Epstein system was done by Ni and Tang^25^, who demonstrated mathematically and experimentally that the system does not admit non-constant steady states if the initial reactant concentrations, reactor size, or effective diffusion rate are not large enough. Mahdy, A. M. investigated the existence and uniqueness of the glioblastoma multiforme (GBM) and IS interaction models^26^, Khader, M. M., et. al., considered the fractional PDEs for the numerical investigation^27^. The approximate analytical solutions for the time-fractional Fokkerâ€“ Planck equation (TFFPE) are obtained^28^ by Mahdy, A. M. Also financial models are investigated using the Caputoâ€“ Fabrizio derivative^29^. Gepreel, K. A., used the algebraic computational methods for the space–time fractional symmetric regularized long wave (SRLW equation), and the space–time fractional coupled Sakharovâ€“ Kuznetsov (Sâ€“ K) equations^30^ and fractional nonlinear Rubella ailment disease model are investigated by the numerical technique^31,32^. In this study, they obtained the precise criteria on the system parameters to guarantee that the spatial homogeneous equilibrium and the spatial homogeneous periodic solutions are Turing-unstable or diffusively unstable. Through the use of a particular Lyapunov functional, Yi et al. demonstrated in 2009 that the constant equilibrium solution is globally asymptotically stable^33^. The Lengyel-Epstein system’s dynamics are examined in Lisena’s 2014 study, which also lowers the standards for the steady-state solution’s global asymptotic stability^34^. In 2013, Wang et al. employed numerical illustrations to illustrate the bifurcation and the Hopf bifurcation theorem to pinpoint the prerequisites for the stability of the equilibrium point. Diffusion-driven instability and bifurcation in the Lengyelâ€“ Epstein system are given by Yi et al. in 2008^35^. Computer simulations of three-dimensional Turing patterns in the Lengyel-Epstein model are given by Shoji and Ohta in 2015^36^. Synchronization results for a class of fractional-order spatiotemporal partial differential systems based on the fractional Lyapunov approach are given by Quannas et al. in 2019^37^. Hopf bifurcations in Lengyel Epstein reactionâ€“ diffusion model with discrete time delay is given by Merdan in 2015^38^. Bifurcations and pattern formation in a generalized Lengyelâ€“ Epstein reaction–diffusion model is given by Mansouri et al. in 2020^39^.

The novelty of this work is that the Lengyelâ€“ Epstein reactionâ€“ diffusion model is considered analytically for the very first time. The generalized Riccati equation mapping technique is applied and gains the different types of wave structures analytically. In the Lengyel-Epstein system, solitary waves can propagate at various rates. The harmony of the system’s diffusive and reactive effects frequently governs the speed of a single wave. Solitary waves can move at a variety of speeds depending on the factors and reaction kinetics. Another important aspect of a single wave is its breadth and amplitude. These are reliant on the reaction kinetics, activator and inhibitor concentrations, and diffusion coefficients. Solitary waves might be more dispersed and have a lower amplitude, or they can be quite localized and have a reasonably high amplitude. Solitary waves are capable of intricate interactions with one another. They can either reject one another or combine to generate larger solo waves, passing past each other unchanged. The specifics of the Lengyel-Epstein model determine the nature of the interaction. A disturbance in the system can lead to the appearance of these solitary waves. They can develop spontaneously or as a result of outside influences, and they frequently do so in areas where the activator concentration has momentarily increased. In this study, we find some new analytical wave structures by using the generalized Riccati equation mapping method.

### The generalized Riccati equation mapping method

The generalized Riccati equation mapping method is define in the following steps^40–43^.

### Step I

Given nonlinear partial differential equation (NPDE) having independent variables $x=(t,x,y,z)$ and dependent variable $w$

1 $$P(w,{w}_{t},{w}_{x},{w}_{y},{w}_{z},{w}_{xx},{w}_{zz},{w}_{xy},{w}_{tt}\cdots )=0,$$

where $P$ is generally a polynomial function of its argument, and the subscripts of dependent variable denotes the partial derivatives.

### Step II

By using the wave transformation, Eq. (1) have the following ansatz

2 $$w=W(\eta ), where\, \eta =x+y-\omega t,$$

where $\eta$ is a real function to be determined. Substituting Eqs. (2) into (1) then we get an ordinary differential equation (ODE) as

3 $$Q(W,{W}_{\eta },{W}_{\eta \eta },\cdots )=0.$$

### Step III

Suppose that the solution of Eq. (3) is in the polynomial form

4 $$W(\eta )=\sum_{j=0}^{n}{a}_{j}\phi (\eta {)}^{j},$$

where ${a}_{j}$ are constants that are determine later and $n$ is positive integer that is obtained by the help of balancing principle. The $\phi (\eta )$ represents the solution of the given generalized riccati equation.

5 $${\phi }^{\mathrm{^{\prime}}}(\eta )=\tau +\rho \phi (\eta )+\chi {\phi }^{2}(\eta ),$$

where $\tau$, $\rho$ and $\chi$ are all real constants. Substituting the Eqs. (4) with (5) into the regarding ODE and remove all the coefficients of $\phi$ will obtain a system of algebraic equations, from which we can get the parameters ${a}_{j},=(j=1,\cdots ,n)$ and $\eta$. Solving the algebraic equations, with the known solutions of Eq. (4), one can be easily obtain the non-travelling wave solutions to the NPDE Eq. (1). We can obtain the following twenty seven solutions to Eq. (3) such as

**Type 1:** For ${\rho }^{2}-4\tau \chi >0$ and $\rho \tau \ne 0$,$(or\rho \chi \ne 0)$ the solutions of Eq. (5) are,

6 $${\phi }_{1}=-\frac{1}{2\chi }\left[\rho +\sqrt{{\rho }^{2}-4\tau \chi }\mathrm{tanh}\left(\frac{\sqrt{{\rho }^{2}-4\tau \chi }}{2}\eta \right)\right],$$

7 $${\phi }_{2}=-\frac{1}{2\chi }\left[\rho +\sqrt{{\rho }^{2}-4\tau \chi }\mathrm{coth}\left(\frac{\sqrt{{\rho }^{2}-4\tau \chi }}{2}\eta \right)\right],$$

8 $${\phi }_{3}=-\frac{1}{2\chi }[\rho +\sqrt{{\rho }^{2}-4\tau \chi }(\mathrm{tanh}(\sqrt{{\rho }^{2}-4\tau \chi }\eta )\pm \mathrm{sec}h(\sqrt{{\rho }^{2}-4\tau \chi }\eta ))],$$

9 $${\phi }_{4}=-\frac{1}{2\chi }[\rho +\sqrt{{\rho }^{2}-4\tau \chi }(\mathrm{coth}(\sqrt{{\rho }^{2}-4\tau \chi }\eta )\pm \mathrm{csc}h(\sqrt{{\rho }^{2}-4\tau \chi }\eta ))],$$

10 $${\phi }_{5}=-\frac{1}{4\chi }[2\rho +\sqrt{{\rho }^{2}-4\tau \chi }\left(\mathrm{tanh}\left(\frac{\sqrt{{\rho }^{2}-4\tau \chi }}{4}\eta \right)\pm \mathrm{coth}\left(\frac{\sqrt{{\rho }^{2}-4\tau \chi }}{4}\eta \right)\right)],$$

11 $${\phi }_{6}=\frac{1}{2\chi }\left[-\rho +\frac{\sqrt{({A}^{2}+{B}^{2})({\rho }^{2}-4\tau \chi )}-A\sqrt{{\rho }^{2}-4\tau \chi }\mathrm{cosh}(\sqrt{{\rho }^{2}-4\tau \chi }\eta )}{A\mathrm{sinh}(\sqrt{{\rho }^{2}-4\tau \chi }\eta )+B}\right],$$

12 $${\phi }_{7}=\frac{1}{2\chi }\left[-\rho -\frac{\sqrt{({A}^{2}+{B}^{2})({\rho }^{2}-4\tau \chi )}+A\sqrt{{\rho }^{2}-4\tau \chi }\mathrm{cosh}(\sqrt{{\rho }^{2}-4\tau \chi }\eta )}{A\mathrm{sinh}(\sqrt{{\rho }^{2}-4\tau \chi }\eta )+B}\right],$$

where $A$ and $B$ are two non zero real constants and satisfies ${B}^{2}-{A}^{2}>0$.

13 $${\phi }_{8}=\frac{2\chi \mathrm{cosh}\left(\frac{\sqrt{{\rho }^{2}-4\tau \chi }}{2}\eta \right)}{\sqrt{{\rho }^{2}-4\tau \chi }\mathrm{sinh}\left(\frac{\sqrt{{\rho }^{2}-4\tau \chi }}{2}\eta \right)-\rho \mathrm{cosh}\left(\frac{\sqrt{{\rho }^{2}-4\tau \chi }}{2}\eta \right)},$$

14 $${\phi }_{9}=\frac{-2\chi \mathrm{sinh}\left(\frac{\sqrt{{\rho }^{2}-4\tau \chi }}{2}\eta \right)}{\rho \mathrm{sinh}\left(\frac{\sqrt{{\rho }^{2}-4\tau \chi }}{2}\eta \right)-\sqrt{{\rho }^{2}-4\tau \chi }\mathrm{cosh}\left(\frac{\sqrt{{\rho }^{2}-4\tau \chi }}{2}\eta \right)},$$

15 $${\phi }_{10}=\frac{2\chi \mathrm{cosh}\left(\frac{\sqrt{{\rho }^{2}-4\tau \chi }}{2}\eta \right)}{\sqrt{{\rho }^{2}-4\tau \chi }\mathrm{sinh}(\sqrt{{\rho }^{2}-4\tau \chi }\eta )-\rho \mathrm{cosh}(\sqrt{{\rho }^{2}-4\tau \chi }\eta )\pm i\sqrt{{\rho }^{2}-4\tau \chi }},$$

16 $${\phi }_{11}=\frac{2\chi \mathrm{sinh}\left(\frac{\sqrt{{\rho }^{2}-4\tau \chi }}{2}\eta \right)}{-\rho \mathrm{sinh}(\sqrt{{\rho }^{2}-4\tau \chi }\eta )+\sqrt{{\rho }^{2}-4\tau \chi }\mathrm{cosh}(\sqrt{{\rho }^{2}-4\tau \chi }\eta )\pm \sqrt{{\rho }^{2}-4\tau \chi }},$$

17 $${\phi }_{12}=\frac{4\chi \mathrm{sinh}\left(\frac{\sqrt{{\rho }^{2}-4\tau \chi }}{4}\eta \right)\mathrm{cosh}\left(\frac{\sqrt{{\rho }^{2}-4\tau \chi }}{4}\eta \right)}{-2\rho \mathrm{sinh}\left(\frac{\sqrt{{\rho }^{2}-4\tau \chi }}{4}\eta \right)\mathrm{cosh}\left(\frac{\sqrt{{\rho }^{2}-4\tau \chi }}{4}\eta \right)+2\sqrt{{\rho }^{2}-4\tau \chi }{\mathrm{cosh}}^{2}\left(\frac{\sqrt{{\rho }^{2}-4\tau \chi }}{4}\eta \right)-\sqrt{{\rho }^{2}-4\tau \chi }}.$$

**Type 2:** when ${\rho }^{2}-4\tau \chi <0$ and $\rho \tau \ne 0$, $(or\tau \chi \ne 0)$ the solutions of Eq. $(5)$ are,

18 $${\phi }_{13}=\frac{1}{2\chi }\left[-\rho +\sqrt{4\tau \chi -{\rho }^{2}}\mathrm{tan}\left(\frac{\sqrt{4\tau \chi -{\rho }^{2}}}{2}\eta \right)\right],$$

19 $${\phi }_{14}=\frac{-1}{2\chi }\left[\rho +\sqrt{4\tau \chi -{\rho }^{2}}\mathrm{cot}\left(\frac{\sqrt{4\tau \chi -{\rho }^{2}}}{2}\eta \right)\right],$$

20 $${\phi }_{15}=\frac{1}{2\chi }\left[-\rho +\sqrt{4\tau \chi -{\rho }^{2}}(\mathrm{tan}(\sqrt{4\tau \chi -{\rho }^{2}}\eta )\pm \mathrm{sec}(\sqrt{4\tau \chi -{\rho }^{2}}\eta ))\right],$$

21 $${\phi }_{16}=\frac{-1}{2\chi }\left[\rho +\sqrt{4\tau \chi -{\rho }^{2}}(\mathrm{cot}(\sqrt{4\tau \chi -{\rho }^{2}}\eta )\pm \mathrm{csc}(\sqrt{4\tau \chi -{\rho }^{2}}\eta ))\right],$$

22 $${\phi }_{17}=\frac{1}{4\chi }\left[-2\rho +\sqrt{4\tau \chi -{\rho }^{2}}\left(\left(\mathrm{tan}\left(\frac{\sqrt{4\tau \chi -{\rho }^{2}}}{4}\eta \right)\right)-\mathrm{cot}\left(\frac{\sqrt{4\tau \chi -{\rho }^{2}}}{4}\eta \right)\right)\right],$$

23 $${\phi }_{18}=\frac{1}{2\chi }\left[-\rho +\frac{\pm \sqrt{({A}^{2}-{B}^{2})(4\tau \chi -{\rho }^{2})}-A\sqrt{4\tau \chi -{\rho }^{2}}\mathrm{cos}(\sqrt{4\tau \chi -{\rho }^{2}}\eta )}{A\mathrm{sin}(\sqrt{4\tau \chi -{\rho }^{2}}\eta )+B}\right],$$

24 $${\phi }_{19}=\frac{1}{2\chi }\left[-\rho -\frac{\pm \sqrt{({A}^{2}-{B}^{2})(4\tau \chi -{\rho }^{2})}+A\sqrt{4\tau \chi -{\rho }^{2}}\mathrm{cos}(\sqrt{4\tau \chi -{\rho }^{2}}\eta )}{A\mathrm{sin}(\sqrt{4\tau \chi -{\rho }^{2}}\eta )+B}\right],$$

where $A$ and $B$ are two non zero real constants and satisfies ${A}^{2}-{B}^{2}>0$.

25 $${\phi }_{20}=-\frac{2\chi \mathrm{cos}\left(\frac{\sqrt{4\tau \chi -{\rho }^{2}}}{2}\eta \right)}{\sqrt{4\tau \chi -{\rho }^{2}}\mathrm{sin}\left(\frac{\sqrt{4\tau \chi -{\rho }^{2}}}{2}\eta \right)+\tau \mathrm{cos}\left(\frac{\sqrt{4\tau \chi -{\rho }^{2}}}{2}\eta \right)},$$

26 $${\phi }_{21}=\frac{2\chi \mathrm{sin}\left(\frac{\sqrt{4\tau \chi -{\rho }^{2}}}{2}\eta \right)}{-\tau \mathrm{sin}\left(\frac{\sqrt{4\tau \chi -{\rho }^{2}}}{2}\eta \right)+\sqrt{4\tau \chi -{\rho }^{2}}\mathrm{cos}\left(\frac{\sqrt{4\tau \chi -{\rho }^{2}}}{2}\eta \right)},$$

27 $${\phi }_{22}=-\frac{2\chi \mathrm{cos}\left(\frac{\sqrt{{\rho }^{2}-4\tau \chi }}{2}\eta \right)}{\sqrt{4\tau \chi -{\rho }^{2}}\mathrm{sin}(\sqrt{4\tau \chi -{\rho }^{2}}\eta )+\tau \mathrm{cos}(\sqrt{4\tau \chi -{\rho }^{2}}\eta )\pm i\sqrt{4\tau \chi -{\rho }^{2}}},$$

28 $${\phi }_{23}=\frac{2\chi \mathrm{sin}\left(\frac{\sqrt{4\tau \chi -{\rho }^{2}}}{2}\eta \right)}{-\rho \mathrm{sin}(\sqrt{4\tau \chi -{\rho }^{2}}\eta )+\sqrt{4\tau \chi -{\rho }^{2}}\mathrm{cos}(\sqrt{4\tau \chi -{\rho }^{2}}\eta )\pm \sqrt{4\tau \chi -{\rho }^{2}}},$$

29 $${\phi }_{24}=\frac{4\chi \mathrm{sin}\left(\frac{\sqrt{4\tau \chi -{\rho }^{2}}}{4}\eta \right)\mathrm{cos}\left(\frac{\sqrt{4\tau \chi -{\rho }^{2}}}{4}\eta \right)}{-2\rho \mathrm{sin}\left(\frac{\sqrt{4\tau \chi -{\rho }^{2}}}{4}\eta \right)\mathrm{cos}\left(\frac{\sqrt{4\tau \chi -{\rho }^{2}}}{4}\eta \right)+2\sqrt{4\tau \chi -{\rho }^{2}}{\mathrm{cos}}^{2}\left(\frac{\sqrt{4\tau \chi -{\rho }^{2}}}{4}\eta \right)-\sqrt{4\tau \chi -{\rho }^{2}}}.$$

**Type 3:** when $\rho =0$ and $\chi \tau \ne 0$, the solutions of Eq. $(5)$ are,

30 $${\phi }_{25}=\frac{-\rho b}{\tau [b+\mathrm{cosh}(\rho \eta )-\mathrm{sinh}(\rho \eta )]},$$

31 $${\phi }_{26}=-\frac{\rho [\mathrm{cosh}(\rho \eta )+\mathrm{sinh}(\rho \eta )]}{\tau [b+\mathrm{cosh}(\rho \eta )+\mathrm{sinh}(\rho \eta )]},$$

where $b$ is any arbitrary constant.

**Type 4:** When $\tau \ne 0$ and $\chi =\rho =0$, the solutions of Eq. $(5)$ are,

32 $${\phi }_{27}=-\frac{1}{\tau \eta +{c}_{1}},$$

where ${c}_{1}$ is an arbitrary constant.

## Application to Lengyel-Epstein reaction diffusion system

In this section, we investigate the analytical solutions of the Lengyel-Epstein system by using the generalized Riccati equation mapping method. Here we illustrate this couple system to achieve the analytical solution of the Lengyel-Epstein system^33,35,45,46^

33 $${u}_{t}=\Delta (u)+a-u-\frac{4uv}{1+{u}^{2}},$$

34 $${v}_{t}=\sigma [c\Delta (v)+b(u-\frac{uv}{1+{u}^{2}})].$$

where $u$ and $v$ are the concentration of the inhibitor chlorite and the activator iodide, respectively. $a$, $b$ and $c$ are the constants. By the wave transformation Eq. (2) we convert the Eqs. (33) and (34) onto the ODEs as follows

35 $$\omega {U}^{\mathrm{^{\prime}}}-2{U}^{\mathrm{^{\prime}}{\prime}}-a+U+\frac{4UV}{1+{U}^{2}}=0,$$

36 $$\omega {V}^{\mathrm{^{\prime}}}-2\sigma c{V}^{\mathrm{^{\prime}}{\prime}}-b(U-\frac{UV}{1+{U}^{2}})=0.$$

Now, we suppose that the solution of Eqs. (35) and (36) as

37 $$U(\xi )=\sum_{i=0}^{N}{\sigma }_{i}{\Omega }^{i}(\xi ),$$

38 $$V(\xi )=\sum_{i=0}^{N}{\delta }_{i}{\Omega }^{i}(\xi ).$$

So, it is satisfies the auxiliary ODE as

39 $${\Omega }^{\mathrm{^{\prime}}}(\xi )=\tau +\rho\Omega (\xi )+\chi\Omega (\xi {)}^{2},$$

Now substituting the value of $N$ by using the homogeneous balancing

40 $$U(\xi )={\sigma }_{0}+{\sigma }_{1}\Omega (\eta ),$$

41 $$V(\xi )={\delta }_{0}+{\delta }_{1}\Omega (\eta )+{\delta }_{2}\Omega (\eta {)}^{2}.$$

By finding the derivatives of the Eqs. (40) and (41) along with Eq. (39) and putting in the Eqs. (35) and (36), and get the system of equations. After solving the system of equation we get the solutions as follows;

**Case 1:** Form the Eq. (35) we gain ${\sigma }_{0}=-\frac{{\delta }_{1}}{\sqrt{4{\delta }_{0}{\delta }_{2}-{\delta }_{1}^{2}}}$, ${\sigma }_{1}=-\frac{2{\delta }_{2}}{\sqrt{4{\delta }_{0}{\delta }_{2}-{\delta }_{1}^{2}}}$, $\omega =\frac{2{\delta }_{2}{\rho }^{2}+{\delta }_{1}^{2}-4{\delta }_{0}{\delta }_{2}-{\delta }_{2}}{{\delta }_{2}\rho }$,

$a=\frac{}{{\delta }_{2}}\left(-\frac{2{\delta }_{2}{\delta }_{1}^{2}\tau }{\sqrt{4{\delta }_{0}{\delta }_{2}-{\delta }_{1}^{2}}\rho }+\frac{8{\delta }_{0}{\delta }_{2}^{2}\tau }{\sqrt{4{\delta }_{0}{\delta }_{2}-{\delta }_{1}^{2}}\rho }+\frac{2{\delta }_{2}^{2}\tau }{\sqrt{4{\delta }_{0}{\delta }_{2}-{\delta }_{1}^{2}}\rho }+\frac{{\delta }_{1}^{3}}{\sqrt{4{\delta }_{0}{\delta }_{2}-{\delta }_{1}^{2}}}-\frac{4{\delta }_{0}{\delta }_{2}{\delta }_{1}}{\sqrt{4{\delta }_{0}{\delta }_{2}-{\delta }_{1}^{2}}}-\frac{{\delta }_{2}{\delta }_{1}}{\sqrt{4{\delta }_{0}{\delta }_{2}-{\delta }_{1}^{2}}}\right).$ For the Eq. (36) we gain ${\delta }_{0}={\sigma }_{0}^{2}+1$, ${\delta }_{1}=2{\sigma }_{0}{\sigma }_{1}$, ${\delta }_{2}={\sigma }_{1}^{2}$, $\rho =\frac{{\sigma }_{1}\tau }{{\sigma }_{0}}$, $\omega =\frac{4c\sigma {\sigma }_{1}\tau }{{\sigma }_{0}}$.

**Type 1:** When ${\rho }^{2}-4\tau \chi >0$ and $\rho \chi \ne 0(or\tau \chi \ne 0)$, we obtained hyperbolic solutions. Substituting the values of constants in Eqs. (40) and (41) and by the help of general solution that are mentioned in methodology we obtained the different form of solutions.

By putting constant values along with Eq. (6) and wave transformation Eq. (2) in the Eqs. (40) and (41) we get the soliton solutions of Eqs. (33) and (34) such as

$${u}_{1}(x,y,t)=-\frac{{\delta }_{1}}{\sqrt{4{\delta }_{0}{\delta }_{2}-{\delta }_{1}^{2}}}+\frac{{\delta }_{2}\left(\rho +\sqrt{{\rho }^{2}-4\tau \chi }\mathrm{tanh}\left(\frac{1}{2}\sqrt{{\rho }^{2}-4\tau \chi }\left(\frac{t\left(2{\delta }_{2}{\rho }^{2}+{\delta }_{1}^{2}-4{\delta }_{0}{\delta }_{2}-{\delta }_{2}\right)}{{\delta }_{2}\rho }+x+y\right)\right)\right)}{\sqrt{4{\delta }_{0}{\delta }_{2}-{\delta }_{1}^{2}}\chi },$$

$${v}_{1}(x,y,t)={\sigma }_{0}^{2}+1+\frac{{\sigma }_{1}^{2}}{4{\chi }^{2}}{\left(\sqrt{\frac{{\sigma }_{1}^{2}{\tau }^{2}}{{\sigma }_{0}^{2}}-4\tau \chi }\mathrm{tanh}\left(\frac{1}{2}\sqrt{\frac{{\sigma }_{1}^{2}{\tau }^{2}}{{\sigma }_{0}^{2}}-4\tau \chi }\left(\frac{4c\sigma {\sigma }_{1}t\tau }{{\sigma }_{0}}+x+y\right)\right)+\frac{{\sigma }_{1}\tau }{{\sigma }_{0}}\right)}^{2}$$

$$-\frac{{\sigma }_{1}{\sigma }_{0}}{\chi }\left(\sqrt{\frac{{\sigma }_{1}^{2}{\tau }^{2}}{{\sigma }_{0}^{2}}-4\tau \chi }\mathrm{tanh}\left(\frac{1}{2}\sqrt{\frac{{\sigma }_{1}^{2}{\tau }^{2}}{{\sigma }_{0}^{2}}-4\tau \chi }\left(\frac{4c\sigma {\sigma }_{1}t\tau }{{\sigma }_{0}}+x+y\right)\right)+\frac{{\sigma }_{1}\tau }{{\sigma }_{0}}\right).$$

By putting constant values along with Eq. (7) and wave transformation Eq. (2) in the Eqs. (40) and (41) we get the soliton solutions of Eqs. (33) and (34) such as

$${u}_{2}(x,y,t)=-\frac{{\delta }_{1}}{\sqrt{4{\delta }_{0}{\delta }_{2}-{\delta }_{1}^{2}}}+\frac{{\delta }_{2}\left(\rho +\sqrt{{\rho }^{2}-4\tau \chi }\mathrm{coth}\left(\frac{1}{2}\sqrt{{\rho }^{2}-4\tau \chi }\left(\frac{t\left(2{\delta }_{2}{\rho }^{2}+{\delta }_{1}^{2}-4{\delta }_{0}{\delta }_{2}-{\delta }_{2}\right)}{{\delta }_{2}\rho }+x+y\right)\right)\right)}{\sqrt{4{\delta }_{0}{\delta }_{2}-{\delta }_{1}^{2}}\chi },$$

$${v}_{2}(x,y,t)={\sigma }_{0}^{2}+1+\frac{{\sigma }_{1}^{2}}{4{\chi }^{2}}{\left(\sqrt{\frac{{\sigma }_{1}^{2}{\tau }^{2}}{{\sigma }_{0}^{2}}-4\tau \chi }\mathrm{coth}\left(\frac{1}{2}\sqrt{\frac{{\sigma }_{1}^{2}{\tau }^{2}}{{\sigma }_{0}^{2}}-4\tau \chi }\left(\frac{4c\sigma {\sigma }_{1}t\tau }{{\sigma }_{0}}+x+y\right)\right)+\frac{{\sigma }_{1}\tau }{{\sigma }_{0}}\right)}^{2}$$

$$-\frac{{\sigma }_{1}{\sigma }_{0}}{\chi }\left(\sqrt{\frac{{\sigma }_{1}^{2}{\tau }^{2}}{{\sigma }_{0}^{2}}-4\tau \chi }\mathrm{coth}\left(\frac{1}{2}\sqrt{\frac{{\sigma }_{1}^{2}{\tau }^{2}}{{\sigma }_{0}^{2}}-4\tau \chi }\left(\frac{4c\sigma {\sigma }_{1}t\tau }{{\sigma }_{0}}+x+y\right)\right)+\frac{{\sigma }_{1}\tau }{{\sigma }_{0}}\right).$$

By putting constant values along with Eq. (8) and wave transformation Eq. (2) in the Eqs. (40) and (41) we get the soliton solutions of Eqs. (33) and (34) such as

$${u}_{3}(x,y,t)=-\frac{{\delta }_{1}}{\sqrt{4{\delta }_{0}{\delta }_{2}-{\delta }_{1}^{2}}}+\frac{{\delta }_{2}}{\sqrt{4{\delta }_{0}{\delta }_{2}-{\delta }_{1}^{2}}\chi }\left(\rho +\sqrt{{\rho }^{2}-4\tau \chi }\left(\mathrm{tanh}\left(\sqrt{{\rho }^{2}-4\tau \chi }\right.\right.\right.$$

$$\left.\left.\left.\times \left(\frac{t\left(2{\delta }_{2}{\rho }^{2}+{\delta }_{1}^{2}-4{\delta }_{0}{\delta }_{2}-{\delta }_{2}\right)}{{\delta }_{2}\rho }+x+y\right)\right)+i\mathrm{sech}\left(\sqrt{{\rho }^{2}-4\tau \chi }\left(\frac{t\left(2{\delta }_{2}{\rho }^{2}+{\delta }_{1}^{2}-4{\delta }_{0}{\delta }_{2}-{\delta }_{2}\right)}{{\delta }_{2}\rho }+x+y\right)\right)\right)\right),$$

$${v}_{3}(x,y,t)={\sigma }_{0}^{2}+1+\frac{{\sigma }_{1}^{2}}{4{\chi }^{2}}\left(\frac{{\sigma }_{1}\tau }{{\sigma }_{0}}+\sqrt{\frac{{\sigma }_{1}^{2}{\tau }^{2}}{{\sigma }_{0}^{2}}-4\tau \chi }\left(\mathrm{tanh}\left(\sqrt{\frac{{\sigma }_{1}^{2}{\tau }^{2}}{{\sigma }_{0}^{2}}-4\tau \chi }\left(\frac{4c\sigma {\sigma }_{1}t\tau }{{\sigma }_{0}}+x+y\right)\right)\right.\right.$$

$${\left.\left.+i\mathrm{sech}\left(\sqrt{\frac{{\sigma }_{1}^{2}{\tau }^{2}}{{\sigma }_{0}^{2}}-4\tau \chi }\left(\frac{4c\sigma {\sigma }_{1}t\tau }{{\sigma }_{0}}+x+y\right)\right)\right)\right)}^{2}-\frac{{\sigma }_{1}{\sigma }_{0}}{\chi }\left(\frac{{\sigma }_{1}\tau }{{\sigma }_{0}}+\sqrt{\frac{{\sigma }_{1}^{2}{\tau }^{2}}{{\sigma }_{0}^{2}}-4\tau \chi }\right.$$

$$\left.\left(\mathrm{tanh}\left(\sqrt{\frac{{\sigma }_{1}^{2}{\tau }^{2}}{{\sigma }_{0}^{2}}-4\tau \chi }\left(\frac{4c\sigma {\sigma }_{1}t\tau }{{\sigma }_{0}}+x+y\right)\right)+i\mathrm{sech}\left(\sqrt{\frac{{\sigma }_{1}^{2}{\tau }^{2}}{{\sigma }_{0}^{2}}-4\tau \chi }\left(\frac{4c\sigma {\sigma }_{1}t\tau }{{\sigma }_{0}}+x+y\right)\right)\right)\right).$$

By putting constant values along with Eq. (9) and wave transformation Eq. (2) in the Eqs. (40) and (41) we get the soliton solutions of Eqs. (33) and (34) such as

$${u}_{4}(x,y,t)=-\frac{{\delta }_{1}}{\sqrt{4{\delta }_{0}{\delta }_{2}-{\delta }_{1}^{2}}}+\frac{{\delta }_{2}}{\sqrt{4{\delta }_{0}{\delta }_{2}-{\delta }_{1}^{2}}\chi }\left(\rho +\sqrt{{\rho }^{2}-4\tau \chi }\left(\mathrm{coth}\left(\sqrt{{\rho }^{2}-4\tau \chi }\right.\right.\right.$$

$$\left.\left.\left.\left(\frac{t\left(2{\delta }_{2}{\rho }^{2}+{\delta }_{1}^{2}-4{\delta }_{0}{\delta }_{2}-{\delta }_{2}\right)}{{\delta }_{2}\rho }+x+y\right)\right)+\mathrm{csch}\left(\sqrt{{\rho }^{2}-4\tau \chi }\left(\frac{t\left(2{\delta }_{2}{\rho }^{2}+{\delta }_{1}^{2}-4{\delta }_{0}{\delta }_{2}-{\delta }_{2}\right)}{{\delta }_{2}\rho }+x+y\right)\right)\right)\right),$$

$${v}_{4}(x,y,t)={\sigma }_{0}^{2}+1-\frac{{\sigma }_{1}{\sigma }_{0}}{\chi }\left(\sqrt{\frac{{\sigma }_{1}^{2}{\tau }^{2}}{{\sigma }_{0}^{2}}-4\tau \chi }\left(\mathrm{coth}\left(\sqrt{\frac{{\sigma }_{1}^{2}{\tau }^{2}}{{\sigma }_{0}^{2}}-4\tau \chi }\left(\frac{4c\sigma {\sigma }_{1}t\tau }{{\sigma }_{0}}+x+y\right)\right)\right.\right.$$

$$\left.\left.+\mathrm{csch}\left(\sqrt{\frac{{\sigma }_{1}^{2}{\tau }^{2}}{{\sigma }_{0}^{2}}-4\tau \chi }\left(\frac{4c\sigma {\sigma }_{1}t\tau }{{\sigma }_{0}}+x+y\right)\right)\right)+\frac{{\sigma }_{1}\tau }{{\sigma }_{0}}\right)+\frac{{\sigma }_{1}^{2}}{4{\chi }^{2}}\left(\sqrt{\frac{{\sigma }_{1}^{2}{\tau }^{2}}{{\sigma }_{0}^{2}}-4\tau \chi }\right.$$

$${\left.\left(\mathrm{coth}\left(\sqrt{\frac{{\sigma }_{1}^{2}{\tau }^{2}}{{\sigma }_{0}^{2}}-4\tau \chi }\left(\frac{4c\sigma {\sigma }_{1}t\tau }{{\sigma }_{0}}+x+y\right)\right)+\mathrm{csch}\left(\sqrt{\frac{{\sigma }_{1}^{2}{\tau }^{2}}{{\sigma }_{0}^{2}}-4\tau \chi }\left(\frac{4c\sigma {\sigma }_{1}t\tau }{{\sigma }_{0}}+x+y\right)\right)\right)+\frac{{\sigma }_{1}\tau }{{\sigma }_{0}}\right)}^{2}.$$

By putting constant values along with Eq. (10) and wave transformation Eq. (2) in the Eqs. (40) and (41) we get the soliton solutions of Eqs. (33) and (34) such as

$${u}_{5}(x,y,t)=-\frac{{\delta }_{1}}{\sqrt{4{\delta }_{0}{\delta }_{2}-{\delta }_{1}^{2}}}+\frac{{\delta }_{2}}{2\sqrt{4{\delta }_{0}{\delta }_{2}-{\delta }_{1}^{2}}\chi }\left(2\rho +\sqrt{{\rho }^{2}-4\tau \chi }\left(\mathrm{tanh}\left(\frac{1}{4}\sqrt{{\rho }^{2}-4\tau \chi }\right.\right.\right.$$

$$\left.\left.\left.\left(\frac{t\left(2{\delta }_{2}{\rho }^{2}+{\delta }_{1}^{2}-4{\delta }_{0}{\delta }_{2}-{\delta }_{2}\right)}{{\delta }_{2}\rho }+x+y\right)\right)+\mathrm{coth}\left(\frac{1}{4}\sqrt{{\rho }^{2}-4\tau \chi }\left(\frac{t\left(2{\delta }_{2}{\rho }^{2}+{\delta }_{1}^{2}-4{\delta }_{0}{\delta }_{2}-{\delta }_{2}\right)}{{\delta }_{2}\rho }+x+y\right)\right)\right)\right),$$

$${v}_{5}(x,y,t)={\sigma }_{0}^{2}+1+\frac{2{\sigma }_{1}\tau }{{\sigma }_{0}}-\frac{{\sigma }_{1}{\sigma }_{0}}{2\chi }\left(\sqrt{\frac{{\sigma }_{1}^{2}{\tau }^{2}}{{\sigma }_{0}^{2}}-4\tau \chi }\left(\mathrm{tanh}\left(\frac{1}{4}\sqrt{\frac{{\sigma }_{1}^{2}{\tau }^{2}}{{\sigma }_{0}^{2}}-4\tau \chi }\left(\frac{4c\sigma {\sigma }_{1}t\tau }{{\sigma }_{0}}+x+y\right)\right)\right.\right.$$

$$\left.\left.+\mathrm{coth}\left(\frac{1}{4}\sqrt{\frac{{\sigma }_{1}^{2}{\tau }^{2}}{{\sigma }_{0}^{2}}-4\tau \chi }\left(\frac{4c\sigma {\sigma }_{1}t\tau }{{\sigma }_{0}}+x+y\right)\right)\right)+\frac{2{\sigma }_{1}\tau }{{\sigma }_{0}}\right)+\frac{{\sigma }_{1}^{2}}{16{\chi }^{2}}\left(\sqrt{\frac{{\sigma }_{1}^{2}{\tau }^{2}}{{\sigma }_{0}^{2}}-4\tau \chi }\right.$$

$${\left.\left(\mathrm{tanh}\left(\frac{1}{4}\sqrt{\frac{{\sigma }_{1}^{2}{\tau }^{2}}{{\sigma }_{0}^{2}}-4\tau \chi }\left(\frac{4c\sigma {\sigma }_{1}t\tau }{{\sigma }_{0}}+x+y\right)\right)+\mathrm{coth}\left(\frac{1}{4}\sqrt{\frac{{\sigma }_{1}^{2}{\tau }^{2}}{{\sigma }_{0}^{2}}-4\tau \chi }\left(\frac{4c\sigma {\sigma }_{1}t\tau }{{\sigma }_{0}}+x+y\right)\right)\right)\right)}^{2}.$$

By putting constant values along with Eq. (11) and wave transformation Eq. (2) in the Eqs. (40) and (41) we get the soliton solutions of Eqs. (33) and (34) such as

$${u}_{6}(x,y,t)=-\frac{{\delta }_{1}}{\sqrt{4{\delta }_{0}{\delta }_{2}-{\delta }_{1}^{2}}}-\frac{{\delta }_{2}}{\sqrt{4{\delta }_{0}{\delta }_{2}-{\delta }_{1}^{2}}\chi }$$

$$\left(\frac{\sqrt{\left({G}^{2}+{H}^{2}\right)\left({\rho }^{2}-4\tau \chi \right)}-G\sqrt{{\rho }^{2}-4\tau \chi }\mathrm{cosh}\left(\sqrt{{\rho }^{2}-4\tau \chi }\left(\frac{t\left(2{\delta }_{2}{\rho }^{2}+{\delta }_{1}^{2}-4{\delta }_{0}{\delta }_{2}-{\delta }_{2}\right)}{{\delta }_{2}\rho }+x+y\right)\right)}{G\mathrm{sinh}\left(\sqrt{{\rho }^{2}-4\tau \chi }\left(\frac{t\left(2{\delta }_{2}{\rho }^{2}+{\delta }_{1}^{2}-4{\delta }_{0}{\delta }_{2}-{\delta }_{2}\right)}{{\delta }_{2}\rho }+x+y\right)\right)+H}-\rho \right),$$

$${v}_{6}(x,y,t)={\sigma }_{0}^{2}+1+\frac{{\sigma }_{1}^{2}}{4{\chi }^{2}}\left(\frac{\sqrt{\left({G}^{2}+{H}^{2}\right)\left(\frac{{\sigma }_{1}^{2}{\tau }^{2}}{{\sigma }_{0}^{2}}-4\tau \chi \right)}-G\sqrt{\frac{{\sigma }_{1}^{2}{\tau }^{2}}{{\sigma }_{0}^{2}}-4\tau \chi }\mathrm{cosh}\left(\sqrt{\frac{{\sigma }_{1}^{2}{\tau }^{2}}{{\sigma }_{0}^{2}}-4\tau \chi }\left(\frac{4c\sigma {\sigma }_{1}t\tau }{{\sigma }_{0}}+x+y\right)\right)}{G\mathrm{sinh}\left(\sqrt{\frac{{\sigma }_{1}^{2}{\tau }^{2}}{{\sigma }_{0}^{2}}-4\tau \chi }\left(\frac{4c\sigma {\sigma }_{1}t\tau }{{\sigma }_{0}}+x+y\right)\right)+H}\right.$$

$${\left.-\frac{{\sigma }_{1}\tau }{{\sigma }_{0}}\right)}^{2}+\frac{{\sigma }_{1}{\sigma }_{0}}{\chi }\left(\frac{\sqrt{\left({G}^{2}+{H}^{2}\right)\left(\frac{{\sigma }_{1}^{2}{\tau }^{2}}{{\sigma }_{0}^{2}}-4\tau \chi \right)}-G\sqrt{\frac{{\sigma }_{1}^{2}{\tau }^{2}}{{\sigma }_{0}^{2}}-4\tau \chi }\mathrm{cosh}\left(\sqrt{\frac{{\sigma }_{1}^{2}{\tau }^{2}}{{\sigma }_{0}^{2}}-4\tau \chi }\left(\frac{4c\sigma {\sigma }_{1}t\tau }{{\sigma }_{0}}+x+y\right)\right)}{G\mathrm{sinh}\left(\sqrt{\frac{{\sigma }_{1}^{2}{\tau }^{2}}{{\sigma }_{0}^{2}}-4\tau \chi }\left(\frac{4c\sigma {\sigma }_{1}t\tau }{{\sigma }_{0}}+x+y\right)\right)+H}-\frac{{\sigma }_{1}\tau }{{\sigma }_{0}}\right).$$

By putting constant values along with Eq. (12) and wave transformation Eq. (2) in the Eqs. (40) and (41) we get the soliton solutions of Eqs. (33) and (34) such as

$${u}_{7}(x,y,t)=-\frac{{\delta }_{1}}{\sqrt{4{\delta }_{0}{\delta }_{2}-{\delta }_{1}^{2}}}-\frac{{\delta }_{2}}{\sqrt{4{\delta }_{0}{\delta }_{2}-{\delta }_{1}^{2}}\chi }$$

$$\left(-\frac{\sqrt{\left({H}^{2}-{G}^{2}\right)\left({\rho }^{2}-4\tau \chi \right)}+G\sqrt{{\rho }^{2}-4\tau \chi }\mathrm{cosh}\left(\sqrt{{\rho }^{2}-4\tau \chi }\left(\frac{t\left(2{\delta }_{2}{\rho }^{2}+{\delta }_{1}^{2}-4{\delta }_{0}{\delta }_{2}-{\delta }_{2}\right)}{{\delta }_{2}\rho }+x+y\right)\right)}{G\mathrm{sinh}\left(\sqrt{{\rho }^{2}-4\tau \chi }\left(\frac{t\left(2{\delta }_{2}{\rho }^{2}+{\delta }_{1}^{2}-4{\delta }_{0}{\delta }_{2}-{\delta }_{2}\right)}{{\delta }_{2}\rho }+x+y\right)\right)+H}-\rho \right),$$

$${v}_{7}(x,y,t)=\frac{{\sigma }_{1}^{2}{\left(-\frac{G\sqrt{\frac{{\sigma }_{1}^{2}{\tau }^{2}}{{\sigma }_{0}^{2}}-4\tau \chi }\mathrm{cosh}\left(\sqrt{\frac{{\sigma }_{1}^{2}{\tau }^{2}}{{\sigma }_{0}^{2}}-4\tau \chi }\left(\frac{4c\sigma {\sigma }_{1}t\tau }{{\sigma }_{0}}+x+y\right)\right)+\sqrt{\left({H}^{2}-{G}^{2}\right)\left(\frac{{\sigma }_{1}^{2}{\tau }^{2}}{{\sigma }_{0}^{2}}-4\tau \chi \right)}}{G\mathrm{sinh}\left(\sqrt{\frac{{\sigma }_{1}^{2}{\tau }^{2}}{{\sigma }_{0}^{2}}-4\tau \chi }\left(\frac{4c\sigma {\sigma }_{1}t\tau }{{\sigma }_{0}}+x+y\right)\right)+H}-\frac{{\sigma }_{1}\tau }{{\sigma }_{0}}\right)}^{2}}{4{\chi }^{2}}$$

$$+\frac{{\sigma }_{1}{\sigma }_{0}\left(-\frac{G\sqrt{\frac{{\sigma }_{1}^{2}{\tau }^{2}}{{\sigma }_{0}^{2}}-4\tau \chi }\mathrm{cosh}\left(\sqrt{\frac{{\sigma }_{1}^{2}{\tau }^{2}}{{\sigma }_{0}^{2}}-4\tau \chi }\left(\frac{4c\sigma {\sigma }_{1}t\tau }{{\sigma }_{0}}+x+y\right)\right)+\sqrt{\left({H}^{2}-{G}^{2}\right)\left(\frac{{\sigma }_{1}^{2}{\tau }^{2}}{{\sigma }_{0}^{2}}-4\tau \chi \right)}}{G\mathrm{sinh}\left(\sqrt{\frac{{\sigma }_{1}^{2}{\tau }^{2}}{{\sigma }_{0}^{2}}-4\tau \chi }\left(\frac{4c\sigma {\sigma }_{1}t\tau }{{\sigma }_{0}}+x+y\right)\right)+H}-\frac{{\sigma }_{1}\tau }{{\sigma }_{0}}\right)}{\chi }+{\sigma }_{0}^{2}+1.$$

By putting constant values along with Eq. (13) and wave transformation Eq. (2) in the Eqs. (40) and (41) we get the soliton solutions of Eqs. (33) and (34) such as

$${u}_{8}(x,y,t)=-\frac{{\delta }_{1}}{\sqrt{4{\delta }_{0}{\delta }_{2}-{\delta }_{1}^{2}}}-\frac{4{\delta }_{2}\chi \mathrm{cosh}\left(\frac{1}{2}\sqrt{{\rho }^{2}-4\tau \chi }\left(\frac{t\left(2{\delta }_{2}{\rho }^{2}+{\delta }_{1}^{2}-4{\delta }_{0}{\delta }_{2}-{\delta }_{2}\right)}{{\delta }_{2}\rho }+x+y\right)\right)}{\sqrt{4{\delta }_{0}{\delta }_{2}-{\delta }_{1}^{2}}\left(\sqrt{{\rho }^{2}-4\tau \chi }\mathrm{sinh}\left(Z\right)-\rho \mathrm{cosh}\left(Z\right)\right)},$$

where $$Z=\frac{1}{2}\sqrt{{\rho }^{2}-4\tau \chi }\left(\frac{t\left(2{\delta }_{2}{\rho }^{2}+{\delta }_{1}^{2}-4{\delta }_{0}{\delta }_{2}-{\delta }_{2}\right)}{{\delta }_{2}\rho }+x+y\right)$$.

$${v}_{8}(x,y,t)={\sigma }_{0}^{2}+1+\frac{4{\sigma }_{1}{\sigma }_{0}\chi \mathrm{cosh}\left(\frac{1}{2}\sqrt{\frac{{\sigma }_{1}^{2}{\tau }^{2}}{{\sigma }_{0}^{2}}-4\tau \chi }\left(\frac{4c\sigma {\sigma }_{1}t\tau }{{\sigma }_{0}}+x+y\right)\right)}{\sqrt{\frac{{\sigma }_{1}^{2}{\tau }^{2}}{{\sigma }_{0}^{2}}-4\tau \chi }\mathrm{sinh}\left(\frac{1}{2}\sqrt{\frac{{\sigma }_{1}^{2}{\tau }^{2}}{{\sigma }_{0}^{2}}-4\tau \chi }\left(\frac{4c\sigma {\sigma }_{1}t\tau }{{\sigma }_{0}}+x+y\right)\right)-\frac{{\sigma }_{1}\tau }{{\sigma }_{0}}\mathrm{cosh}\left(Z\right)}$$

$$+\frac{4{\sigma }_{1}^{2}{\chi }^{2}{\mathrm{cosh}}^{2}\left(\frac{1}{2}\sqrt{\frac{{\sigma }_{1}^{2}{\tau }^{2}}{{\sigma }_{0}^{2}}-4\tau \chi }\left(\frac{4c\sigma {\sigma }_{1}t\tau }{{\sigma }_{0}}+x+y\right)\right)}{{\left(\sqrt{\frac{{\sigma }_{1}^{2}{\tau }^{2}}{{\sigma }_{0}^{2}}-4\tau \chi }\mathrm{sinh}\left(\frac{1}{2}\sqrt{\frac{{\sigma }_{1}^{2}{\tau }^{2}}{{\sigma }_{0}^{2}}-4\tau \chi }\left(\frac{4c\sigma {\sigma }_{1}t\tau }{{\sigma }_{0}}+x+y\right)\right)-\frac{{\sigma }_{1}\tau }{{\sigma }_{0}}\mathrm{cosh}\left(Z\right)\right)}^{2}},$$

where $Z=\frac{1}{2}\sqrt{\frac{{\sigma }_{1}^{2}{\tau }^{2}}{{\sigma }_{0}^{2}}-4\tau \chi }\left(\frac{4c\sigma {\sigma }_{1}t\tau }{{\sigma }_{0}}+x+y\right)$. By putting constant values along with Eq. (14) and wave transformation Eq. (2) in the Eqs. (40) and (41) we get the soliton solutions of Eqs. (33) and (34) such as

$${u}_{9}(x,y,t)=-\frac{{\delta }_{1}}{\sqrt{4{\delta }_{0}{\delta }_{2}-{\delta }_{1}^{2}}}+\frac{4{\delta }_{2}\chi \mathrm{sinh}\left(\frac{1}{2}\sqrt{{\rho }^{2}-4\tau \chi }\left(\frac{t\left(2{\delta }_{2}{\rho }^{2}+{\delta }_{1}^{2}-4{\delta }_{0}{\delta }_{2}-{\delta }_{2}\right)}{{\delta }_{2}\rho }+x+y\right)\right)}{\sqrt{4{\delta }_{0}{\delta }_{2}-{\delta }_{1}^{2}}\left(\rho \mathrm{sinh}\left(Z\right)-\sqrt{{\rho }^{2}-4\tau \chi }\mathrm{cosh}\left(Z\right)\right)},$$

where $$Z=\frac{1}{2}\sqrt{{\rho }^{2}-4\tau \chi }\left(\frac{t\left(2{\delta }_{2}{\rho }^{2}+{\delta }_{1}^{2}-4{\delta }_{0}{\delta }_{2}-{\delta }_{2}\right)}{{\delta }_{2}\rho }+x+y\right)$$.

$${v}_{9}(x,y,t)={\sigma }_{0}^{2}+1-\frac{4{\sigma }_{1}{\sigma }_{0}\chi \mathrm{sinh}\left(\frac{1}{2}\sqrt{\frac{{\sigma }_{1}^{2}{\tau }^{2}}{{\sigma }_{0}^{2}}-4\tau \chi }\left(\frac{4c\sigma {\sigma }_{1}t\tau }{{\sigma }_{0}}+x+y\right)\right)}{\frac{{\sigma }_{1}\tau \mathrm{sinh}\left(\frac{1}{2}\sqrt{\frac{{\sigma }_{1}^{2}{\tau }^{2}}{{\sigma }_{0}^{2}}-4\tau \chi }\left(\frac{4c\sigma {\sigma }_{1}t\tau }{{\sigma }_{0}}+x+y\right)\right)}{{\sigma }_{0}}-\sqrt{\frac{{\sigma }_{1}^{2}{\tau }^{2}}{{\sigma }_{0}^{2}}-4\tau \chi }\mathrm{cosh}\left(Z\right)}$$

$$+\frac{4{\sigma }_{1}^{2}{\chi }^{2}{\mathrm{sinh}}^{2}\left(\frac{1}{2}\sqrt{\frac{{\sigma }_{1}^{2}{\tau }^{2}}{{\sigma }_{0}^{2}}-4\tau \chi }\left(\frac{4c\sigma {\sigma }_{1}t\tau }{{\sigma }_{0}}+x+y\right)\right)}{{\left(\frac{{\sigma }_{1}\tau \mathrm{sinh}\left(\frac{1}{2}\sqrt{\frac{{\sigma }_{1}^{2}{\tau }^{2}}{{\sigma }_{0}^{2}}-4\tau \chi }\left(\frac{4c\sigma {\sigma }_{1}t\tau }{{\sigma }_{0}}+x+y\right)\right)}{{\sigma }_{0}}-\sqrt{\frac{{\sigma }_{1}^{2}{\tau }^{2}}{{\sigma }_{0}^{2}}-4\tau \chi }\mathrm{cosh}\left(Z\right)\right)}^{2}},$$

where $Z=\frac{1}{2}\sqrt{\frac{{\sigma }_{1}^{2}{\tau }^{2}}{{\sigma }_{0}^{2}}-4\tau \chi }\left(\frac{4c\sigma {\sigma }_{1}t\tau }{{\sigma }_{0}}+x+y\right)$. By putting constant values along with Eq. (15) and wave transformation Eq. (2) in the Eqs. (40) and (41) we get the soliton solutions of Eqs. (33) and (34) such as

$${u}_{10}(x,y,t)=-\frac{{\delta }_{1}}{\sqrt{4{\delta }_{0}{\delta }_{2}-{\delta }_{1}^{2}}}-\frac{4{\delta }_{2}\chi \mathrm{cosh}\left(\sqrt{{\rho }^{2}-4\tau \chi }\left(\frac{t\left(2{\delta }_{2}{\rho }^{2}+{\delta }_{1}^{2}-4{\delta }_{0}{\delta }_{2}-{\delta }_{2}\right)}{{\delta }_{2}\rho }+x+y\right)\right)}{\sqrt{4{\delta }_{0}{\delta }_{2}-{\delta }_{1}^{2}}\left(-i\sqrt{{\rho }^{2}-4\tau \chi }+\sqrt{{\rho }^{2}-4\tau \chi }\mathrm{sinh}\left(Z\right)-\rho \mathrm{cosh}\left(Z\right)\right)},$$

where $$Z=\sqrt{{\rho }^{2}-4\tau \chi }\left(\frac{t\left(2{\delta }_{2}{\rho }^{2}+{\delta }_{1}^{2}-4{\delta }_{0}{\delta }_{2}-{\delta }_{2}\right)}{{\delta }_{2}\rho }+x+y\right)$$.

$${v}_{10}(x,y,t)={\sigma }_{0}^{2}+1+\frac{4{\sigma }_{1}{\sigma }_{0}\chi \mathrm{cosh}\left(\sqrt{\frac{{\sigma }_{1}^{2}{\tau }^{2}}{{\sigma }_{0}^{2}}-4\tau \chi }\left(\frac{4c\sigma {\sigma }_{1}t\tau }{{\sigma }_{0}}+x+y\right)\right)}{\sqrt{\frac{{\sigma }_{1}^{2}{\tau }^{2}}{{\sigma }_{0}^{2}}-4\tau \chi }\mathrm{sinh}\left(\sqrt{\frac{{\sigma }_{1}^{2}{\tau }^{2}}{{\sigma }_{0}^{2}}-4\tau \chi }\left(\frac{4c\sigma {\sigma }_{1}t\tau }{{\sigma }_{0}}+x+y\right)\right)-\frac{{\sigma }_{1}\tau \mathrm{cosh}\left(Z\right)}{{\sigma }_{0}}-i\sqrt{\frac{{\sigma }_{1}^{2}{\tau }^{2}}{{\sigma }_{0}^{2}}-4\tau \chi }}$$

$$+\frac{4{\sigma }_{1}^{2}{\chi }^{2}{\mathrm{cosh}}^{2}\left(\sqrt{\frac{{\sigma }_{1}^{2}{\tau }^{2}}{{\sigma }_{0}^{2}}-4\tau \chi }\left(\frac{4c\sigma {\sigma }_{1}t\tau }{{\sigma }_{0}}+x+y\right)\right)}{{\left(\sqrt{\frac{{\sigma }_{1}^{2}{\tau }^{2}}{{\sigma }_{0}^{2}}-4\tau \chi }\mathrm{sinh}\left(Z\right)-\frac{{\sigma }_{1}\tau \mathrm{cosh}\left(Z\right)}{{\sigma }_{0}}-i\sqrt{\frac{{\sigma }_{1}^{2}{\tau }^{2}}{{\sigma }_{0}^{2}}-4\tau \chi }\right)}^{2}},$$

where $Z=\sqrt{\frac{{\sigma }_{1}^{2}{\tau }^{2}}{{\sigma }_{0}^{2}}-4\tau \chi }\left(\frac{4c\sigma {\sigma }_{1}t\tau }{{\sigma }_{0}}+x+y\right)$. By putting constant values along with Eq. (16) and wave transformation Eq. (2) in the Eqs. (40) and (41) we get the soliton solutions of Eqs. (33) and (34) such as

$${u}_{11}(x,y,t)=-\frac{{\delta }_{1}}{\sqrt{4{\delta }_{0}{\delta }_{2}-{\delta }_{1}^{2}}}-\frac{4{\delta }_{2}\chi \mathrm{sinh}\left(\sqrt{{\rho }^{2}-4\tau \chi }\left(\frac{t\left(2{\delta }_{2}{\rho }^{2}+{\delta }_{1}^{2}-4{\delta }_{0}{\delta }_{2}-{\delta }_{2}\right)}{{\delta }_{2}\rho }+x+y\right)\right)}{\sqrt{4{\delta }_{0}{\delta }_{2}-{\delta }_{1}^{2}}\left(\sqrt{{\rho }^{2}-4\tau \chi }-\rho \mathrm{sinh}\left(Z\right)+\sqrt{{\rho }^{2}-4\tau \chi }\mathrm{cosh}\left(Z\right)\right)},$$

where $$Z=\sqrt{{\rho }^{2}-4\tau \chi }\left(\frac{t\left(2{\delta }_{2}{\rho }^{2}+{\delta }_{1}^{2}-4{\delta }_{0}{\delta }_{2}-{\delta }_{2}\right)}{{\delta }_{2}\rho }+x+y\right)$$.

$${v}_{11}(x,y,t)={\sigma }_{0}^{2}+1+\frac{4{\sigma }_{1}{\sigma }_{0}\chi \mathrm{sinh}\left(\sqrt{\frac{{\sigma }_{1}^{2}{\tau }^{2}}{{\sigma }_{0}^{2}}-4\tau \chi }\left(\frac{4c\sigma {\sigma }_{1}t\tau }{{\sigma }_{0}}+x+y\right)\right)}{-\frac{{\sigma }_{1}\tau \mathrm{sinh}\left(\sqrt{\frac{{\sigma }_{1}^{2}{\tau }^{2}}{{\sigma }_{0}^{2}}-4\tau \chi }\left(\frac{4c\sigma {\sigma }_{1}t\tau }{{\sigma }_{0}}+x+y\right)\right)}{{\sigma }_{0}}+\sqrt{\frac{{\sigma }_{1}^{2}{\tau }^{2}}{{\sigma }_{0}^{2}}-4\tau \chi }\mathrm{cosh}\left(Z\right)+\sqrt{\frac{{\sigma }_{1}^{2}{\tau }^{2}}{{\sigma }_{0}^{2}}-4\tau \chi }}$$

$$+\frac{4{\sigma }_{1}^{2}{\chi }^{2}{\mathrm{sinh}}^{2}\left(\sqrt{\frac{{\sigma }_{1}^{2}{\tau }^{2}}{{\sigma }_{0}^{2}}-4\tau \chi }\left(\frac{4c\sigma {\sigma }_{1}t\tau }{{\sigma }_{0}}+x+y\right)\right)}{{\left(-\frac{{\sigma }_{1}\tau \mathrm{sinh}\left(Z\right)}{{\sigma }_{0}}+\sqrt{\frac{{\sigma }_{1}^{2}{\tau }^{2}}{{\sigma }_{0}^{2}}-4\tau \chi }\mathrm{cosh}\left(Z\right)+\sqrt{\frac{{\sigma }_{1}^{2}{\tau }^{2}}{{\sigma }_{0}^{2}}-4\tau \chi }\right)}^{2}},$$

where $Z=\sqrt{\frac{{\sigma }_{1}^{2}{\tau }^{2}}{{\sigma }_{0}^{2}}-4\tau \chi }\left(\frac{4c\sigma {\sigma }_{1}t\tau }{{\sigma }_{0}}+x+y\right)$. By putting constant values along with Eq. (17) and wave transformation Eq. (2) in the Eqs. (40) and (41) we get the soliton solutions of Eqs. (33) and (34) such as

$${u}_{12}(x,y,t)=-\frac{{\delta }_{1}}{\sqrt{4{\delta }_{0}{\delta }_{2}-{\delta }_{1}^{2}}}-\frac{8{\delta }_{2}\chi \mathrm{sinh}\left(\frac{1}{4}\sqrt{{\rho }^{2}-4\tau \chi }\left(\frac{t\left(2{\delta }_{2}{\rho }^{2}+{\delta }_{1}^{2}-4{\delta }_{0}{\delta }_{2}-{\delta }_{2}\right)}{{\delta }_{2}\rho }+x+y\right)\right)\mathrm{cosh}\left(Z\right)}{\sqrt{4{\delta }_{0}{\delta }_{2}-{\delta }_{1}^{2}}\left(-\sqrt{{\rho }^{2}-4\tau \chi }+2\sqrt{{\rho }^{2}-4\tau \chi }{\mathrm{cosh}}^{2}\left(Z\right)-2\rho \mathrm{sinh}\left(Z\right)\mathrm{cosh}\left(Z\right)\right)},$$

where $$Z=\frac{1}{4}\sqrt{{\rho }^{2}-4\tau \chi }\left(\frac{t\left(2{\delta }_{2}{\rho }^{2}+{\delta }_{1}^{2}-4{\delta }_{0}{\delta }_{2}-{\delta }_{2}\right)}{{\delta }_{2}\rho }+x+y\right)$$.

$${v}_{12}(x,y,t)={\sigma }_{0}^{2}+1+\frac{16{\sigma }_{1}^{2}{\chi }^{2}{\mathrm{sinh}}^{2}\left(\frac{1}{4}\sqrt{\frac{{\sigma }_{1}^{2}{\tau }^{2}}{{\sigma }_{0}^{2}}-4\tau \chi }\left(\frac{4c\sigma {\sigma }_{1}t\tau }{{\sigma }_{0}}+x+y\right)\right){\mathrm{cosh}}^{2}\left(Z\right)}{{\left(2\sqrt{\frac{{\sigma }_{1}^{2}{\tau }^{2}}{{\sigma }_{0}^{2}}-4\tau \chi }{\mathrm{cosh}}^{2}\left(Z\right)-\frac{2{\sigma }_{1}\tau \mathrm{sinh}\left(Z\right)\mathrm{cosh}\left(Z\right)}{{\sigma }_{0}}-\sqrt{\frac{{\sigma }_{1}^{2}{\tau }^{2}}{{\sigma }_{0}^{2}}-4\tau \chi }\right)}^{2}}$$

$$+\frac{8{\sigma }_{0}{\sigma }_{1}\chi \mathrm{sinh}\left(\frac{1}{4}\sqrt{\frac{{\sigma }_{1}^{2}{\tau }^{2}}{{\sigma }_{0}^{2}}-4\tau \chi }\left(\frac{4c\sigma {\sigma }_{1}t\tau }{{\sigma }_{0}}+x+y\right)\right)\mathrm{cosh}\left(Z\right)}{2\sqrt{\frac{{\sigma }_{1}^{2}{\tau }^{2}}{{\sigma }_{0}^{2}}-4\tau \chi }{\mathrm{cosh}}^{2}\left(Z\right)-\frac{2{\sigma }_{1}\tau \mathrm{sinh}\left(Z\right)\mathrm{cosh}\left(\frac{1}{4}\sqrt{\frac{{\sigma }_{1}^{2}{\tau }^{2}}{{\sigma }_{0}^{2}}-4\tau \chi }\left(\frac{4c\sigma {\sigma }_{1}t\tau }{{\sigma }_{0}}+x+y\right)\right)}{{\sigma }_{0}}-\sqrt{\frac{{\sigma }_{1}^{2}{\tau }^{2}}{{\sigma }_{0}^{2}}-4\tau \chi }},$$

where $$Z=\frac{1}{4}\sqrt{\frac{{\sigma }_{1}^{2}{\tau }^{2}}{{\sigma }_{0}^{2}}-4\tau \chi }\left(\frac{4c\sigma {\sigma }_{1}t\tau }{{\sigma }_{0}}+x+y\right)$$.

**Type 2:** When ${\tau }^{2}-4\rho \chi <0$ and $\tau \rho \ne 0(or\tau \chi \ne 0)$, we obtained trigonometric solutions. Substituting the values of constants in Eqs. (40) and (41) and by the help of general solution that are mentioned in methodology we obtained the different form of solutions.

By putting constant values along with Eq. (18) and wave transformation Eq. (2) in the Eqs. (40) and (41) we get the solitary solutions of Eqs. (33) and (34) such as

$${u}_{13}(x,y,t)=-\frac{{\delta }_{1}}{\sqrt{4{\delta }_{0}{\delta }_{2}-{\delta }_{1}^{2}}}-\frac{{\delta }_{2}\left(\sqrt{4\tau \chi -{\rho }^{2}}\mathrm{tan}\left(\frac{1}{2}\sqrt{4\tau \chi -{\rho }^{2}}\left(\frac{t\left(2{\delta }_{2}{\rho }^{2}+{\delta }_{1}^{2}-4{\delta }_{0}{\delta }_{2}-{\delta }_{2}\right)}{{\delta }_{2}\rho }+x+y\right)\right)-\rho \right)}{\sqrt{4{\delta }_{0}{\delta }_{2}-{\delta }_{1}^{2}}\chi },$$

$${v}_{13}(x,y,t)=\frac{{\sigma }_{1}^{2}{\left(\sqrt{4\tau \chi -\frac{{\sigma }_{1}^{2}{\tau }^{2}}{{\sigma }_{0}^{2}}}\mathrm{tan}\left(\frac{1}{2}\sqrt{4\tau \chi -\frac{{\sigma }_{1}^{2}{\tau }^{2}}{{\sigma }_{0}^{2}}}\left(\frac{4c\sigma {\sigma }_{1}t\tau }{{\sigma }_{0}}+x+y\right)\right)-\frac{{\sigma }_{1}\tau }{{\sigma }_{0}}\right)}^{2}}{4{\chi }^{2}}$$

$$+\frac{{\sigma }_{1}{\sigma }_{0}\left(\sqrt{4\tau \chi -\frac{{\sigma }_{1}^{2}{\tau }^{2}}{{\sigma }_{0}^{2}}}\mathrm{tan}\left(\frac{1}{2}\sqrt{4\tau \chi -\frac{{\sigma }_{1}^{2}{\tau }^{2}}{{\sigma }_{0}^{2}}}\left(\frac{4c\sigma {\sigma }_{1}t\tau }{{\sigma }_{0}}+x+y\right)\right)-\frac{{\sigma }_{1}\tau }{{\sigma }_{0}}\right)}{\chi }+{\sigma }_{0}^{2}+1.$$

By putting constant values along with Eq. (19) and wave transformation Eq. (2) in the Eqs. (40) and (41) we get the solitary wave solutions of Eqs. (33) and (34) such as

$${u}_{14}(x,y,t)=-\frac{{\delta }_{1}}{\sqrt{4{\delta }_{0}{\delta }_{2}-{\delta }_{1}^{2}}}+\frac{{\delta }_{2}\left(\rho +\sqrt{4\tau \chi -{\rho }^{2}}\mathrm{cot}\left(\frac{1}{2}\sqrt{4\tau \chi -{\rho }^{2}}\left(\frac{t\left(2{\delta }_{2}{\rho }^{2}+{\delta }_{1}^{2}-4{\delta }_{0}{\delta }_{2}-{\delta }_{2}\right)}{{\delta }_{2}\rho }+x+y\right)\right)\right)}{\sqrt{4{\delta }_{0}{\delta }_{2}-{\delta }_{1}^{2}}\chi },$$

$${v}_{14}(x,y,t)=\frac{{\sigma }_{1}^{2}{\left(\sqrt{4\tau \chi -\frac{{\sigma }_{1}^{2}{\tau }^{2}}{{\sigma }_{0}^{2}}}\mathrm{cot}\left(\frac{1}{2}\sqrt{4\tau \chi -\frac{{\sigma }_{1}^{2}{\tau }^{2}}{{\sigma }_{0}^{2}}}\left(\frac{4c\sigma {\sigma }_{1}t\tau }{{\sigma }_{0}}+x+y\right)\right)+\frac{{\sigma }_{1}\tau }{{\sigma }_{0}}\right)}^{2}}{4{\chi }^{2}}$$

$$-\frac{{\sigma }_{1}{\sigma }_{0}\left(\sqrt{4\tau \chi -\frac{{\sigma }_{1}^{2}{\tau }^{2}}{{\sigma }_{0}^{2}}}\mathrm{cot}\left(\frac{1}{2}\sqrt{4\tau \chi -\frac{{\sigma }_{1}^{2}{\tau }^{2}}{{\sigma }_{0}^{2}}}\left(\frac{4c\sigma {\sigma }_{1}t\tau }{{\sigma }_{0}}+x+y\right)\right)+\frac{{\sigma }_{1}\tau }{{\sigma }_{0}}\right)}{\chi }+{\sigma }_{0}^{2}+1.$$

By putting constant values along with Eq. (20) and wave transformation Eq. (2) in the Eqs. (40) and (41) we get the solitary wave solutions of Eqs. (33) and (34) such as

$${u}_{15}(x,y,t)=-\frac{{\delta }_{1}}{\sqrt{4{\delta }_{0}{\delta }_{2}-{\delta }_{1}^{2}}}-\frac{{\delta }_{2}}{\sqrt{4{\delta }_{0}{\delta }_{2}-{\delta }_{1}^{2}}\chi }\left(-\rho \sqrt{4\tau \chi -{\rho }^{2}}\left(\mathrm{tan}\left(\sqrt{4\tau \chi -{\rho }^{2}}\right.\right.\right.$$

$$\left.\left.\left.\left(\frac{t\left(2{\delta }_{2}{\rho }^{2}+{\delta }_{1}^{2}-4{\delta }_{0}{\delta }_{2}-{\delta }_{2}\right)}{{\delta }_{2}\rho }+x+y\right)\right)-\mathrm{sec}\left(\sqrt{4\tau \chi -{\rho }^{2}}\left(\frac{t\left(2{\delta }_{2}{\rho }^{2}+{\delta }_{1}^{2}-4{\delta }_{0}{\delta }_{2}-{\delta }_{2}\right)}{{\delta }_{2}\rho }+x+y\right)\right)\right)\right),$$

$${v}_{15}(x,y,t)={\sigma }_{0}^{2}+1+\frac{{\sigma }_{1}{\sigma }_{0}}{\chi }\left(\sqrt{4\tau \chi -\frac{{\sigma }_{1}^{2}{\tau }^{2}}{{\sigma }_{0}^{2}}}\left(\mathrm{tan}\left(\sqrt{4\tau \chi -\frac{{\sigma }_{1}^{2}{\tau }^{2}}{{\sigma }_{0}^{2}}}\left(\frac{4c\sigma {\sigma }_{1}t\tau }{{\sigma }_{0}}+x+y\right)\right)\right.\right.$$

$$\left.\left.-\mathrm{sec}\left(\sqrt{4\tau \chi -\frac{{\sigma }_{1}^{2}{\tau }^{2}}{{\sigma }_{0}^{2}}}\left(\frac{4c\sigma {\sigma }_{1}t\tau }{{\sigma }_{0}}+x+y\right)\right)\right)-\frac{{\sigma }_{1}\tau }{{\sigma }_{0}}\right)+\frac{{\sigma }_{1}^{2}}{4{\chi }^{2}}\left(\sqrt{4\tau \chi -\frac{{\sigma }_{1}^{2}{\tau }^{2}}{{\sigma }_{0}^{2}}}\right.$$

$${\left.\left(\mathrm{tan}\left(\sqrt{4\tau \chi -\frac{{\sigma }_{1}^{2}{\tau }^{2}}{{\sigma }_{0}^{2}}}\left(\frac{4c\sigma {\sigma }_{1}t\tau }{{\sigma }_{0}}+x+y\right)\right)-\mathrm{sec}\left(\sqrt{4\tau \chi -\frac{{\sigma }_{1}^{2}{\tau }^{2}}{{\sigma }_{0}^{2}}}\left(\frac{4c\sigma {\sigma }_{1}t\tau }{{\sigma }_{0}}+x+y\right)\right)\right)-\frac{{\sigma }_{1}\tau }{{\sigma }_{0}}\right)}^{2}.$$

By putting constant values along with Eq. (21) and wave transformation Eq. (2) in the Eqs. (40) and (41) we get the solitary wave solutions of Eqs. (33) and (34) such as

$${u}_{16}(x,y,t)=-\frac{{\delta }_{1}}{\sqrt{4{\delta }_{0}{\delta }_{2}-{\delta }_{1}^{2}}}+\frac{{\delta }_{2}}{\sqrt{4{\delta }_{0}{\delta }_{2}-{\delta }_{1}^{2}}\chi }\left(\rho +\sqrt{4\tau \chi -{\rho }^{2}}\left(\mathrm{cot}\left(\sqrt{4\tau \chi -{\rho }^{2}}\right.\right.\right.$$

$$\left.\left.\left.\left(\frac{t\left(2{\delta }_{2}{\rho }^{2}+{\delta }_{1}^{2}-4{\delta }_{0}{\delta }_{2}-{\delta }_{2}\right)}{{\delta }_{2}\rho }+x+y\right)\right)-\mathrm{csc}\left(\sqrt{4\tau \chi -{\rho }^{2}}\left(\frac{t\left(2{\delta }_{2}{\rho }^{2}+{\delta }_{1}^{2}-4{\delta }_{0}{\delta }_{2}-{\delta }_{2}\right)}{{\delta }_{2}\rho }+x+y\right)\right)\right)\right),$$

$${v}_{16}(x,y,t)={\sigma }_{0}^{2}+1-\frac{{\sigma }_{1}{\sigma }_{0}}{\chi }\left(\sqrt{4\tau \chi -\frac{{\sigma }_{1}^{2}{\tau }^{2}}{{\sigma }_{0}^{2}}}\left(\mathrm{cot}\left(\sqrt{4\tau \chi -\frac{{\sigma }_{1}^{2}{\tau }^{2}}{{\sigma }_{0}^{2}}}\left(\frac{4c\sigma {\sigma }_{1}t\tau }{{\sigma }_{0}}+x+y\right)\right)\right.\right.$$

$$\left.\left.-\mathrm{csc}\left(\sqrt{4\tau \chi -\frac{{\sigma }_{1}^{2}{\tau }^{2}}{{\sigma }_{0}^{2}}}\left(\frac{4c\sigma {\sigma }_{1}t\tau }{{\sigma }_{0}}+x+y\right)\right)\right)+\frac{{\sigma }_{1}\tau }{{\sigma }_{0}}\right)+\frac{{\sigma }_{1}^{2}}{4{\chi }^{2}}\left(\sqrt{4\tau \chi -\frac{{\sigma }_{1}^{2}{\tau }^{2}}{{\sigma }_{0}^{2}}}\right.$$

$${\left.\left(\mathrm{cot}\left(\sqrt{4\tau \chi -\frac{{\sigma }_{1}^{2}{\tau }^{2}}{{\sigma }_{0}^{2}}}\left(\frac{4c\sigma {\sigma }_{1}t\tau }{{\sigma }_{0}}+x+y\right)\right)-\mathrm{csc}\left(\sqrt{4\tau \chi -\frac{{\sigma }_{1}^{2}{\tau }^{2}}{{\sigma }_{0}^{2}}}\left(\frac{4c\sigma {\sigma }_{1}t\tau }{{\sigma }_{0}}+x+y\right)\right)\right)+\frac{{\sigma }_{1}\tau }{{\sigma }_{0}}\right)}^{2}.$$

By putting constant values along with Eq. (22) and wave transformation Eq. (2) in the Eqs. (40) and (41) we get the solitary wave solutions of Eqs. (33) and (34) such as

$${u}_{17}(x,y,t)=-\frac{{\delta }_{1}}{\sqrt{4{\delta }_{0}{\delta }_{2}-{\delta }_{1}^{2}}}-\frac{{\delta }_{2}}{2\sqrt{4{\delta }_{0}{\delta }_{2}-{\delta }_{1}^{2}}\chi }\left(\sqrt{4\tau \chi -{\rho }^{2}}\left(\mathrm{tan}\left(\frac{1}{4}\sqrt{4\tau \chi -{\rho }^{2}}\right.\right.\right.$$

$$\left.\left.\left.\left(\frac{t\left(2{\delta }_{2}{\rho }^{2}+{\delta }_{1}^{2}-4{\delta }_{0}{\delta }_{2}-{\delta }_{2}\right)}{{\delta }_{2}\rho }+x+y\right)\right)-\mathrm{cot}\left(\frac{1}{4}\sqrt{4\tau \chi -{\rho }^{2}}\left(\frac{t\left(2{\delta }_{2}{\rho }^{2}+{\delta }_{1}^{2}-4{\delta }_{0}{\delta }_{2}-{\delta }_{2}\right)}{{\delta }_{2}\rho }+x+y\right)\right)\right)-2\rho \right),$$

$${v}_{17}(x,y,t)={\sigma }_{0}^{2}+1+\frac{{\sigma }_{1}{\sigma }_{0}}{2\chi }\left(\sqrt{4\tau \chi -\frac{{\sigma }_{1}^{2}{\tau }^{2}}{{\sigma }_{0}^{2}}}\left(\mathrm{tan}\left(\frac{1}{4}\sqrt{4\tau \chi -\frac{{\sigma }_{1}^{2}{\tau }^{2}}{{\sigma }_{0}^{2}}}\left(\frac{4c\sigma {\sigma }_{1}t\tau }{{\sigma }_{0}}+x+y\right)\right)\right.\right.$$

$$\left.\left.-\mathrm{cot}\left(\frac{1}{4}\sqrt{4\tau \chi -\frac{{\sigma }_{1}^{2}{\tau }^{2}}{{\sigma }_{0}^{2}}}\left(\frac{4c\sigma {\sigma }_{1}t\tau }{{\sigma }_{0}}+x+y\right)\right)\right)-\frac{2{\sigma }_{1}\tau }{{\sigma }_{0}}\right)+\frac{{\sigma }_{1}^{2}}{16{\chi }^{2}}\left(\sqrt{4\tau \chi -\frac{{\sigma }_{1}^{2}{\tau }^{2}}{{\sigma }_{0}^{2}}}\right.$$

$${\left.\left(\mathrm{tan}\left(\frac{1}{4}\sqrt{4\tau \chi -\frac{{\sigma }_{1}^{2}{\tau }^{2}}{{\sigma }_{0}^{2}}}\left(\frac{4c\sigma {\sigma }_{1}t\tau }{{\sigma }_{0}}+x+y\right)\right)-\mathrm{cot}\left(\frac{1}{4}\sqrt{4\tau \chi -\frac{{\sigma }_{1}^{2}{\tau }^{2}}{{\sigma }_{0}^{2}}}\left(\frac{4c\sigma {\sigma }_{1}t\tau }{{\sigma }_{0}}+x+y\right)\right)\right)-\frac{2{\sigma }_{1}\tau }{{\sigma }_{0}}\right)}^{2}.$$

By putting constant values along with Eq. (23) and wave transformation Eq. (2) in the Eqs. (40) and (41) we get the solitary wave solutions of Eqs. (33) and (34) such as

$${u}_{18}(x,y,t)=-\frac{{\delta }_{1}}{\sqrt{4{\delta }_{0}{\delta }_{2}-{\delta }_{1}^{2}}}-\frac{{\delta }_{2}}{\sqrt{4{\delta }_{0}{\delta }_{2}-{\delta }_{1}^{2}}\chi }$$

$$\left(-\rho +\frac{\sqrt{\left({G}^{2}-{H}^{2}\right)\left(4\tau \chi -{\rho }^{2}\right)}-G\sqrt{4\tau \chi -{\rho }^{2}}\mathrm{cos}\left(\sqrt{4\tau \chi -{\rho }^{2}}\left(\frac{t\left(2{\delta }_{2}{\rho }^{2}+{\delta }_{1}^{2}-4{\delta }_{0}{\delta }_{2}-{\delta }_{2}\right)}{{\delta }_{2}\rho }+x+y\right)\right)}{G\mathrm{sin}\left(\sqrt{4\tau \chi -{\rho }^{2}}\left(\frac{t\left(2{\delta }_{2}{\rho }^{2}+{\delta }_{1}^{2}-4{\delta }_{0}{\delta }_{2}-{\delta }_{2}\right)}{{\delta }_{2}\rho }+x+y\right)\right)+H}\right),$$

$${v}_{18}(x,y,t)=\frac{{\sigma }_{1}^{2}{\left(\frac{\sqrt{\left({G}^{2}-{H}^{2}\right)\left(4\tau \chi -\frac{{\sigma }_{1}^{2}{\tau }^{2}}{{\sigma }_{0}^{2}}\right)}-G\sqrt{4\tau \chi -\frac{{\sigma }_{1}^{2}{\tau }^{2}}{{\sigma }_{0}^{2}}}\mathrm{cos}\left(\sqrt{4\tau \chi -\frac{{\sigma }_{1}^{2}{\tau }^{2}}{{\sigma }_{0}^{2}}}\left(\frac{4c\sigma {\sigma }_{1}t\tau }{{\sigma }_{0}}+x+y\right)\right)}{G\mathrm{sin}\left(\sqrt{4\tau \chi -\frac{{\sigma }_{1}^{2}{\tau }^{2}}{{\sigma }_{0}^{2}}}\left(\frac{4c\sigma {\sigma }_{1}t\tau }{{\sigma }_{0}}+x+y\right)\right)+H}-\frac{{\sigma }_{1}\tau }{{\sigma }_{0}}\right)}^{2}}{4{\chi }^{2}}$$

$$+\frac{{\sigma }_{1}{\sigma }_{0}\left(\frac{\sqrt{\left({G}^{2}-{H}^{2}\right)\left(4\tau \chi -\frac{{\sigma }_{1}^{2}{\tau }^{2}}{{\sigma }_{0}^{2}}\right)}-G\sqrt{4\tau \chi -\frac{{\sigma }_{1}^{2}{\tau }^{2}}{{\sigma }_{0}^{2}}}\mathrm{cos}\left(\sqrt{4\tau \chi -\frac{{\sigma }_{1}^{2}{\tau }^{2}}{{\sigma }_{0}^{2}}}\left(\frac{4c\sigma {\sigma }_{1}t\tau }{{\sigma }_{0}}+x+y\right)\right)}{G\mathrm{sin}\left(\sqrt{4\tau \chi -\frac{{\sigma }_{1}^{2}{\tau }^{2}}{{\sigma }_{0}^{2}}}\left(\frac{4c\sigma {\sigma }_{1}t\tau }{{\sigma }_{0}}+x+y\right)\right)+H}-\frac{{\sigma }_{1}\tau }{{\sigma }_{0}}\right)}{\chi }+{\sigma }_{0}^{2}+1.$$

By putting constant values along with Eq. (24) and wave transformation Eq. (2) in the Eqs. (40) and (41) we get the solitary wave solutions of Eqs. (33) and (34) such as

$${u}_{19}(x,y,t)=-\frac{{\delta }_{1}}{\sqrt{4{\delta }_{0}{\delta }_{2}-{\delta }_{1}^{2}}}-\frac{{\delta }_{2}}{\sqrt{4{\delta }_{0}{\delta }_{2}-{\delta }_{1}^{2}}\chi }$$

$$\left(-\frac{\sqrt{\left({G}^{2}-{H}^{2}\right)\left(4\tau \chi -{\rho }^{2}\right)}+G\sqrt{4\tau \chi -{\rho }^{2}}\mathrm{cos}\left(\sqrt{4\tau \chi -{\rho }^{2}}\left(\frac{t\left(2{\delta }_{2}{\rho }^{2}+{\delta }_{1}^{2}-4{\delta }_{0}{\delta }_{2}-{\delta }_{2}\right)}{{\delta }_{2}\rho }+x+y\right)\right)}{G\mathrm{sin}\left(\sqrt{4\tau \chi -{\rho }^{2}}\left(\frac{t\left(2{\delta }_{2}{\rho }^{2}+{\delta }_{1}^{2}-4{\delta }_{0}{\delta }_{2}-{\delta }_{2}\right)}{{\delta }_{2}\rho }+x+y\right)\right)+H}-\rho \right),$$

$${v}_{19}(x,y,t)=\frac{{\sigma }_{1}^{2}{\left(-\frac{G\sqrt{4\tau \chi -\frac{{\sigma }_{1}^{2}{\tau }^{2}}{{\sigma }_{0}^{2}}}\mathrm{cos}\left(\sqrt{4\tau \chi -\frac{{\sigma }_{1}^{2}{\tau }^{2}}{{\sigma }_{0}^{2}}}\left(\frac{4c\sigma {\sigma }_{1}t\tau }{{\sigma }_{0}}+x+y\right)\right)+\sqrt{\left({G}^{2}-{H}^{2}\right)\left(4\tau \chi -\frac{{\sigma }_{1}^{2}{\tau }^{2}}{{\sigma }_{0}^{2}}\right)}}{G\mathrm{sin}\left(\sqrt{4\tau \chi -\frac{{\sigma }_{1}^{2}{\tau }^{2}}{{\sigma }_{0}^{2}}}\left(\frac{4c\sigma {\sigma }_{1}t\tau }{{\sigma }_{0}}+x+y\right)\right)+H}-\frac{{\sigma }_{1}\tau }{{\sigma }_{0}}\right)}^{2}}{4{\chi }^{2}}$$

$$+\frac{{\sigma }_{1}{\sigma }_{0}\left(-\frac{G\sqrt{4\tau \chi -\frac{{\sigma }_{1}^{2}{\tau }^{2}}{{\sigma }_{0}^{2}}}\mathrm{cos}\left(\sqrt{4\tau \chi -\frac{{\sigma }_{1}^{2}{\tau }^{2}}{{\sigma }_{0}^{2}}}\left(\frac{4c\sigma {\sigma }_{1}t\tau }{{\sigma }_{0}}+x+y\right)\right)+\sqrt{\left({G}^{2}-{H}^{2}\right)\left(4\tau \chi -\frac{{\sigma }_{1}^{2}{\tau }^{2}}{{\sigma }_{0}^{2}}\right)}}{G\mathrm{sin}\left(\sqrt{4\tau \chi -\frac{{\sigma }_{1}^{2}{\tau }^{2}}{{\sigma }_{0}^{2}}}\left(\frac{4c\sigma {\sigma }_{1}t\tau }{{\sigma }_{0}}+x+y\right)\right)+H}-\frac{{\sigma }_{1}\tau }{{\sigma }_{0}}\right)}{\chi }+{\sigma }_{0}^{2}+1,$$

where $G$ and $H$ are two non-zero real constants and satisfies ${G}^{2}-{H}^{2}>0$.

By putting constant values along with Eq. (25) and wave transformation Eq. (2) in the Eqs. (40) and (41) we get the solitary wave solutions of Eqs. (33) and (34) such as

42 $${u}_{20}(x,y,t)=-\frac{{\delta }_{1}}{\sqrt{4{\delta }_{0}{\delta }_{2}-{\delta }_{1}^{2}}}+\frac{4{\delta }_{2}\chi \mathrm{cos}\left(\frac{1}{2}\sqrt{4\tau \chi -{\rho }^{2}}\left(\frac{t\left(2{\delta }_{2}{\rho }^{2}+{\delta }_{1}^{2}-4{\delta }_{0}{\delta }_{2}-{\delta }_{2}\right)}{{\delta }_{2}\rho }+x+y\right)\right)}{\sqrt{4{\delta }_{0}{\delta }_{2}-{\delta }_{1}^{2}}\left(\sqrt{4\tau \chi -{\rho }^{2}}\mathrm{sin}\left(Z\right)+\rho \mathrm{cos}\left(Z\right)\right)},$$

where $$Z=\frac{1}{2}\sqrt{4\tau \chi -{\rho }^{2}}\left(\frac{t\left(2{\delta }_{2}{\rho }^{2}+{\delta }_{1}^{2}-4{\delta }_{0}{\delta }_{2}-{\delta }_{2}\right)}{{\delta }_{2}\rho }+x+y\right)$$.

$${v}_{20}(x,y,t)={\sigma }_{0}^{2}+1-\frac{4{\sigma }_{1}{\sigma }_{0}\chi \mathrm{cos}\left(\frac{1}{2}\sqrt{4\tau \chi -\frac{{\sigma }_{1}^{2}{\tau }^{2}}{{\sigma }_{0}^{2}}}\left(\frac{4c\sigma {\sigma }_{1}t\tau }{{\sigma }_{0}}+x+y\right)\right)}{\sqrt{4\tau \chi -\frac{{\sigma }_{1}^{2}{\tau }^{2}}{{\sigma }_{0}^{2}}}\mathrm{sin}\left(Z\right)+\frac{{\sigma }_{1}\tau \mathrm{cos}\left(Z\right)}{{\sigma }_{0}}}$$

$$+\frac{4{\sigma }_{1}^{2}{\chi }^{2}{\mathrm{cos}}^{2}\left(\frac{1}{2}\sqrt{4\tau \chi -\frac{{\sigma }_{1}^{2}{\tau }^{2}}{{\sigma }_{0}^{2}}}\left(\frac{4c\sigma {\sigma }_{1}t\tau }{{\sigma }_{0}}+x+y\right)\right)}{{\left(\sqrt{4\tau \chi -\frac{{\sigma }_{1}^{2}{\tau }^{2}}{{\sigma }_{0}^{2}}}\mathrm{sin}\left(Z\right)+\frac{{\sigma }_{1}\tau \mathrm{cos}\left(\frac{1}{2}\sqrt{4\tau \chi -\frac{{\sigma }_{1}^{2}{\tau }^{2}}{{\sigma }_{0}^{2}}}\left(\frac{4c\sigma {\sigma }_{1}t\tau }{{\sigma }_{0}}+x+y\right)\right)}{{\sigma }_{0}}\right)}^{2}},$$

where $G=\frac{1}{2}\sqrt{4\tau \chi -\frac{{\sigma }_{1}^{2}{\tau }^{2}}{{\sigma }_{0}^{2}}}\left(\frac{4c\sigma {\sigma }_{1}t\tau }{{\sigma }_{0}}+x+y\right)$. By putting constant values along with Eq. (26) and wave transformation Eq. (2) in the Eqs. (40) and (41) we get the solitary wave solutions of Eqs. (33) and (34) such as

$${u}_{21}(x,y,t)=-\frac{{\delta }_{1}}{\sqrt{4{\delta }_{0}{\delta }_{2}-{\delta }_{1}^{2}}}-\frac{4{\delta }_{2}\chi \mathrm{sin}\left(\frac{1}{2}\sqrt{4\tau \chi -{\rho }^{2}}\left(\frac{t\left(2{\delta }_{2}{\rho }^{2}+{\delta }_{1}^{2}-4{\delta }_{0}{\delta }_{2}-{\delta }_{2}\right)}{{\delta }_{2}\rho }+x+y\right)\right)}{\sqrt{4{\delta }_{0}{\delta }_{2}-{\delta }_{1}^{2}}\left(\sqrt{4\tau \chi -{\rho }^{2}}\mathrm{cos}\left(Z\right)-\rho \mathrm{sin}\left(Z\right)\right)},$$

where $$Z=\frac{1}{2}\sqrt{4\tau \chi -{\rho }^{2}}\left(\frac{t\left(2{\delta }_{2}{\rho }^{2}+{\delta }_{1}^{2}-4{\delta }_{0}{\delta }_{2}-{\delta }_{2}\right)}{{\delta }_{2}\rho }+x+y\right)$$.

$${v}_{21}(x,y,t)={\sigma }_{0}^{2}+1+\frac{4{\sigma }_{1}^{2}{\chi }^{2}{\mathrm{sin}}^{2}\left(\frac{1}{2}\sqrt{4\tau \chi -\frac{{\sigma }_{1}^{2}{\tau }^{2}}{{\sigma }_{0}^{2}}}\left(\frac{4c\sigma {\sigma }_{1}t\tau }{{\sigma }_{0}}+x+y\right)\right)}{{\left(\sqrt{4\tau \chi -\frac{{\sigma }_{1}^{2}{\tau }^{2}}{{\sigma }_{0}^{2}}}\mathrm{cos}\left(\frac{1}{2}\sqrt{4\tau \chi -\frac{{\sigma }_{1}^{2}{\tau }^{2}}{{\sigma }_{0}^{2}}}\left(\frac{4c\sigma {\sigma }_{1}t\tau }{{\sigma }_{0}}+x+y\right)\right)-\frac{{\sigma }_{1}\tau \mathrm{sin}\left(Z\right)}{{\sigma }_{0}}\right)}^{2}}$$

$$+\frac{4{\sigma }_{1}{\sigma }_{0}\chi \mathrm{sin}\left(\frac{1}{2}\sqrt{4\tau \chi -\frac{{\sigma }_{1}^{2}{\tau }^{2}}{{\sigma }_{0}^{2}}}\left(\frac{4c\sigma {\sigma }_{1}t\tau }{{\sigma }_{0}}+x+y\right)\right)}{\sqrt{4\tau \chi -\frac{{\sigma }_{1}^{2}{\tau }^{2}}{{\sigma }_{0}^{2}}}\mathrm{cos}\left(\frac{1}{2}\sqrt{4\tau \chi -\frac{{\sigma }_{1}^{2}{\tau }^{2}}{{\sigma }_{0}^{2}}}\left(\frac{4c\sigma {\sigma }_{1}t\tau }{{\sigma }_{0}}+x+y\right)\right)-\frac{{\sigma }_{1}\tau \mathrm{sin}\left(Z\right)}{{\sigma }_{0}}},$$

where $Z=\frac{1}{2}\sqrt{4\tau \chi -\frac{{\sigma }_{1}^{2}{\tau }^{2}}{{\sigma }_{0}^{2}}}\left(\frac{4c\sigma {\sigma }_{1}t\tau }{{\sigma }_{0}}+x+y\right)$. By putting constant values along with Eq. (27) and wave transformation Eq. (2) in the Eqs. (40) and (41) we get the solitary wave solutions of Eqs. (33) and (34) such as

$${u}_{22}(x,y,t)=-\frac{{\delta }_{1}}{\sqrt{4{\delta }_{0}{\delta }_{2}-{\delta }_{1}^{2}}}+\frac{4{\delta }_{2}\chi \mathrm{cos}\left(\sqrt{4\tau \chi -{\rho }^{2}}\left(\frac{t\left(2{\delta }_{2}{\rho }^{2}+{\delta }_{1}^{2}-4{\delta }_{0}{\delta }_{2}-{\delta }_{2}\right)}{{\delta }_{2}\rho }+x+y\right)\right)}{\sqrt{4{\delta }_{0}{\delta }_{2}-{\delta }_{1}^{2}}\left(\sqrt{4\tau \chi -{\rho }^{2}}+\sqrt{4\tau \chi -{\rho }^{2}}\mathrm{sin}\left(Z\right)+\rho \mathrm{cos}\left(Z\right)\right)},$$

where $$Z=\sqrt{4\tau \chi -{\rho }^{2}}\left(\frac{t\left(2{\delta }_{2}{\rho }^{2}+{\delta }_{1}^{2}-4{\delta }_{0}{\delta }_{2}-{\delta }_{2}\right)}{{\delta }_{2}\rho }+x+y\right)$$.

$${v}_{22}(x,y,t)={\sigma }_{0}^{2}+1-\frac{4{\sigma }_{1}{\sigma }_{0}\chi \mathrm{cos}\left(\sqrt{4\tau \chi -\frac{{\sigma }_{1}^{2}{\tau }^{2}}{{\sigma }_{0}^{2}}}\left(\frac{4c\sigma {\sigma }_{1}t\tau }{{\sigma }_{0}}+x+y\right)\right)}{\sqrt{4\tau \chi -\frac{{\sigma }_{1}^{2}{\tau }^{2}}{{\sigma }_{0}^{2}}}\mathrm{sin}\left(\sqrt{4\tau \chi -\frac{{\sigma }_{1}^{2}{\tau }^{2}}{{\sigma }_{0}^{2}}}\left(\frac{4c\sigma {\sigma }_{1}t\tau }{{\sigma }_{0}}+x+y\right)\right)+\frac{{\sigma }_{1}\tau \mathrm{cos}\left(Z\right)}{{\sigma }_{0}}+\sqrt{4\tau \chi -\frac{{\sigma }_{1}^{2}{\tau }^{2}}{{\sigma }_{0}^{2}}}}$$

$$+\frac{4{\sigma }_{1}^{2}{\chi }^{2}{\mathrm{cos}}^{2}\left(\sqrt{4\tau \chi -\frac{{\sigma }_{1}^{2}{\tau }^{2}}{{\sigma }_{0}^{2}}}\left(\frac{4c\sigma {\sigma }_{1}t\tau }{{\sigma }_{0}}+x+y\right)\right)}{{\left(\sqrt{4\tau \chi -\frac{{\sigma }_{1}^{2}{\tau }^{2}}{{\sigma }_{0}^{2}}}\mathrm{sin}\left(\sqrt{4\tau \chi -\frac{{\sigma }_{1}^{2}{\tau }^{2}}{{\sigma }_{0}^{2}}}\left(\frac{4c\sigma {\sigma }_{1}t\tau }{{\sigma }_{0}}+x+y\right)\right)+\frac{{\sigma }_{1}\tau \mathrm{cos}\left(Z\right)}{{\sigma }_{0}}+\sqrt{4\tau \chi -\frac{{\sigma }_{1}^{2}{\tau }^{2}}{{\sigma }_{0}^{2}}}\right)}^{2}},$$

where $Z=\sqrt{4\tau \chi -\frac{{\sigma }_{1}^{2}{\tau }^{2}}{{\sigma }_{0}^{2}}}\left(\frac{4c\sigma {\sigma }_{1}t\tau }{{\sigma }_{0}}+x+y\right)$. By putting constant values along with Eq. (28) and wave transformation Eq. (2) in the Eqs. (40) and (41) we get the solitary wave solutions of Eqs. (33) and (34) such as

$${u}_{23}(x,y,t)=-\frac{{\delta }_{1}}{\sqrt{4{\delta }_{0}{\delta }_{2}-{\delta }_{1}^{2}}}-\frac{4{\delta }_{2}\chi \mathrm{sin}\left(\sqrt{4\tau \chi -{\rho }^{2}}\left(\frac{t\left(2{\delta }_{2}{\rho }^{2}+{\delta }_{1}^{2}-4{\delta }_{0}{\delta }_{2}-{\delta }_{2}\right)}{{\delta }_{2}\rho }+x+y\right)\right)}{\sqrt{4{\delta }_{0}{\delta }_{2}-{\delta }_{1}^{2}}\left(\sqrt{4\tau \chi -{\rho }^{2}}-\rho \mathrm{sin}\left(Z\right)+\sqrt{4\tau \chi -{\rho }^{2}}\mathrm{cos}\left(Z\right)\right)},$$

where $$Z=\sqrt{4\tau \chi -{\rho }^{2}}\left(\frac{t\left(2{\delta }_{2}{\rho }^{2}+{\delta }_{1}^{2}-4{\delta }_{0}{\delta }_{2}-{\delta }_{2}\right)}{{\delta }_{2}\rho }+x+y\right)$$.

$${v}_{23}(x,y,t)={\sigma }_{0}^{2}+1+\frac{4{\sigma }_{1}{\sigma }_{0}\chi \mathrm{sin}\left(\sqrt{4\tau \chi -\frac{{\sigma }_{1}^{2}{\tau }^{2}}{{\sigma }_{0}^{2}}}\left(\frac{4c\sigma {\sigma }_{1}t\tau }{{\sigma }_{0}}+x+y\right)\right)}{-\frac{{\sigma }_{1}\tau \mathrm{sin}\left(Z\right)}{{\sigma }_{0}}+\sqrt{4\tau \chi -\frac{{\sigma }_{1}^{2}{\tau }^{2}}{{\sigma }_{0}^{2}}}\mathrm{cos}\left(\sqrt{4\tau \chi -\frac{{\sigma }_{1}^{2}{\tau }^{2}}{{\sigma }_{0}^{2}}}\left(\frac{4c\sigma {\sigma }_{1}t\tau }{{\sigma }_{0}}+x+y\right)\right)+\sqrt{4\tau \chi -\frac{{\sigma }_{1}^{2}{\tau }^{2}}{{\sigma }_{0}^{2}}}}$$

$$+\frac{4{\sigma }_{1}^{2}{\chi }^{2}{\mathrm{sin}}^{2}\left(\sqrt{4\tau \chi -\frac{{\sigma }_{1}^{2}{\tau }^{2}}{{\sigma }_{0}^{2}}}\left(\frac{4c\sigma {\sigma }_{1}t\tau }{{\sigma }_{0}}+x+y\right)\right)}{{\left(-\frac{{\sigma }_{1}\tau \mathrm{sin}\left(\sqrt{4\tau \chi -\frac{{\sigma }_{1}^{2}{\tau }^{2}}{{\sigma }_{0}^{2}}}\left(\frac{4c\sigma {\sigma }_{1}t\tau }{{\sigma }_{0}}+x+y\right)\right)}{{\sigma }_{0}}+\sqrt{4\tau \chi -\frac{{\sigma }_{1}^{2}{\tau }^{2}}{{\sigma }_{0}^{2}}}\mathrm{cos}\left(Z\right)+\sqrt{4\tau \chi -\frac{{\sigma }_{1}^{2}{\tau }^{2}}{{\sigma }_{0}^{2}}}\right)}^{2}},$$

where $Z=\sqrt{4\tau \chi -\frac{{\sigma }_{1}^{2}{\tau }^{2}}{{\sigma }_{0}^{2}}}\left(\frac{4c\sigma {\sigma }_{1}t\tau }{{\sigma }_{0}}+x+y\right)$. By putting constant values along with Eq. (29) and wave transformation Eq. (2) in the Eqs. (40) and (41) we get the solitary wave solutions of Eqs. (33) and (34) such as

$${u}_{24}(x,y,t)=-\frac{{\delta }_{1}}{\sqrt{4{\delta }_{0}{\delta }_{2}-{\delta }_{1}^{2}}}-\frac{8{\delta }_{2}\chi \mathrm{sin}\left(Z\right)\mathrm{cos}\left(\frac{1}{4}\sqrt{4\tau \chi -{\rho }^{2}}\left(\frac{t\left(2{\delta }_{2}{\rho }^{2}+{\delta }_{1}^{2}-4{\delta }_{0}{\delta }_{2}-{\delta }_{2}\right)}{{\delta }_{2}\rho }+x+y\right)\right)}{\sqrt{4{\delta }_{0}{\delta }_{2}-{\delta }_{1}^{2}}\left(-\sqrt{4\tau \chi -{\rho }^{2}}+2\sqrt{4\tau \chi -{\rho }^{2}}{\mathrm{cos}}^{2}\left(Z\right)-2\rho \mathrm{sin}\left(Z\right)\mathrm{cos}\left(Z\right)\right)},$$

where $$Z=\frac{1}{4}\sqrt{4\tau \chi -{\rho }^{2}}\left(\frac{t\left(2{\delta }_{2}{\rho }^{2}+{\delta }_{1}^{2}-4{\delta }_{0}{\delta }_{2}-{\delta }_{2}\right)}{{\delta }_{2}\rho }+x+y\right)$$.

$${v}_{24}(x,y,t)={\sigma }_{0}^{2}+1+\frac{8{\sigma }_{1}{\sigma }_{0}\chi \mathrm{sin}\left(\frac{1}{4}\sqrt{4\tau \chi -\frac{{\sigma }_{1}^{2}{\tau }^{2}}{{\sigma }_{0}^{2}}}\left(\frac{4c\sigma {\sigma }_{1}t\tau }{{\sigma }_{0}}+x+y\right)\right)\mathrm{cos}\left(Z\right)}{2\sqrt{4\tau \chi -\frac{{\sigma }_{1}^{2}{\tau }^{2}}{{\sigma }_{0}^{2}}}{\mathrm{cos}}^{2}\left(Z\right)-\frac{2{\sigma }_{1}\tau \mathrm{sin}\left(Z\right)\mathrm{cos}\left(Z\right)}{{\sigma }_{0}}-\sqrt{4\tau \chi -\frac{{\sigma }_{1}^{2}{\tau }^{2}}{{\sigma }_{0}^{2}}}}$$

$$+\frac{16{\sigma }_{1}^{2}{\chi }^{2}{\mathrm{sin}}^{2}\left(\frac{1}{4}\sqrt{4\tau \chi -\frac{{\sigma }_{1}^{2}{\tau }^{2}}{{\sigma }_{0}^{2}}}\left(\frac{4c\sigma {\sigma }_{1}t\tau }{{\sigma }_{0}}+x+y\right)\right){\mathrm{cos}}^{2}\left(Z\right)}{{\left(2\sqrt{4\tau \chi -\frac{{\sigma }_{1}^{2}{\tau }^{2}}{{\sigma }_{0}^{2}}}{\mathrm{cos}}^{2}\left(Z\right)-\frac{2{\sigma }_{1}\tau \mathrm{sin}\left(Z\right)\mathrm{cos}\left(Z\right)}{{\sigma }_{0}}-\sqrt{4\tau \chi -\frac{{\sigma }_{1}^{2}{\tau }^{2}}{{\sigma }_{0}^{2}}}\right)}^{2}},$$

where $$Z=\frac{1}{4}\sqrt{4\tau \chi -\frac{{\sigma }_{1}^{2}{\tau }^{2}}{{\sigma }_{0}^{2}}}\left(\frac{4c\sigma {\sigma }_{1}t\tau }{{\sigma }_{0}}+x+y\right)$$.

**Type 3:** When $\chi =0$ and $\tau \rho \ne 0$, we obtained hyperbolic function solutions. By putting constant values along with Eq. (30) and wave transformation Eq. (2) in the Eqs. (40) and (41) we get the hyperbolic function solutions of Eqs. (33) and (34) such as

$${u}_{25}(x,y,t)=-\frac{{\delta }_{1}}{\sqrt{4{\delta }_{0}{\delta }_{2}-{\delta }_{1}^{2}}}+\frac{2d{\delta }_{2}\rho }{\sqrt{4{\delta }_{0}{\delta }_{2}-{\delta }_{1}^{2}}q\left(d-\mathrm{sinh}\left(\rho \left(\frac{t\left(2{\delta }_{2}{\rho }^{2}+{\delta }_{1}^{2}-4{\delta }_{0}{\delta }_{2}-{\delta }_{2}\right)}{{\delta }_{2}\rho }+x+y\right)\right)+\mathrm{cosh}\left(Z\right)\right)},$$

where $$Z=\rho \left(\frac{t\left(2{\delta }_{2}{\rho }^{2}+{\delta }_{1}^{2}-4{\delta }_{0}{\delta }_{2}-{\delta }_{2}\right)}{{\delta }_{2}\rho }+x+y\right)$$.

$${v}_{25}(x,y,t)=\frac{{d}^{2}{\sigma }_{1}^{4}{\tau }^{2}}{{q}^{2}{\sigma }_{0}^{2}{\left(-\mathrm{sinh}\left(\frac{{\sigma }_{1}\tau \left(\frac{4c\sigma {\sigma }_{1}t\tau }{{\sigma }_{0}}+x+y\right)}{{\sigma }_{0}}\right)+\mathrm{cosh}\left(\frac{{\sigma }_{1}\tau \left(\frac{4c\sigma {\sigma }_{1}t\tau }{{\sigma }_{0}}+x+y\right)}{{\sigma }_{0}}\right)+d\right)}^{2}}$$

$$-\frac{2d{\sigma }_{1}^{2}\tau }{q\left(-\mathrm{sinh}\left(\frac{{\sigma }_{1}\tau \left(\frac{4c\sigma {\sigma }_{1}t\tau }{{\sigma }_{0}}+x+y\right)}{{\sigma }_{0}}\right)+\mathrm{cosh}\left(\frac{{\sigma }_{1}\tau \left(\frac{4c\sigma {\sigma }_{1}t\tau }{{\sigma }_{0}}+x+y\right)}{{\sigma }_{0}}\right)+d\right)}+{\sigma }_{0}^{2}+1.$$

By putting constant values along with Eq. (31) and wave transformation Eq. (2) in the Eqs. (40) and (41) we get the hyperbolic function solutions of Eqs. (33) and (34) such as

$${u}_{26}(x,y,t)=-\frac{{\delta }_{1}}{\sqrt{4{\delta }_{0}{\delta }_{2}-{\delta }_{1}^{2}}}-\frac{2{\delta }_{2}\rho \left(\mathrm{sinh}\left(\rho \left(\frac{t\left(2{\delta }_{2}{\rho }^{2}+{\delta }_{1}^{2}-4{\delta }_{0}{\delta }_{2}-{\delta }_{2}\right)}{{\delta }_{2}\rho }+x+y\right)\right)+\mathrm{cosh}\left(Z\right)\right)}{\sqrt{4{\delta }_{0}{\delta }_{2}-{\delta }_{1}^{2}}q\left(d+\mathrm{sinh}\left(\rho \left(\frac{t\left(2{\delta }_{2}{\rho }^{2}+{\delta }_{1}^{2}-4{\delta }_{0}{\delta }_{2}-{\delta }_{2}\right)}{{\delta }_{2}\rho }+x+y\right)\right)+\mathrm{cosh}\left(Z\right)\right)},$$

where $$Z=\rho \left(\frac{t\left(2{\delta }_{2}{\rho }^{2}+{\delta }_{1}^{2}-4{\delta }_{0}{\delta }_{2}-{\delta }_{2}\right)}{{\delta }_{2}\rho }+x+y\right)$$.

$${v}_{26}(x,y,t)=\frac{{\sigma }_{1}^{4}{\tau }^{2}{\left(\mathrm{sinh}\left(\frac{{\sigma }_{1}\tau \left(\frac{4c\sigma {\sigma }_{1}t\tau }{{\sigma }_{0}}+x+y\right)}{{\sigma }_{0}}\right)+\mathrm{cosh}\left(\frac{{\sigma }_{1}\tau \left(\frac{4c\sigma {\sigma }_{1}t\tau }{{\sigma }_{0}}+x+y\right)}{{\sigma }_{0}}\right)\right)}^{2}}{{q}^{2}{\sigma }_{0}^{2}{\left(\mathrm{sinh}\left(\frac{{\sigma }_{1}\tau \left(\frac{4c\sigma {\sigma }_{1}t\tau }{{\sigma }_{0}}+x+y\right)}{{\sigma }_{0}}\right)+\mathrm{cosh}\left(\frac{{\sigma }_{1}\tau \left(\frac{4c\sigma {\sigma }_{1}t\tau }{{\sigma }_{0}}+x+y\right)}{{\sigma }_{0}}\right)+d\right)}^{2}}$$

$$+\frac{2{\sigma }_{1}^{2}\tau \left(\mathrm{sinh}\left(\frac{{\sigma }_{1}\tau \left(\frac{4c\sigma {\sigma }_{1}t\tau }{{\sigma }_{0}}+x+y\right)}{{\sigma }_{0}}\right)+\mathrm{cosh}\left(\frac{{\sigma }_{1}\tau \left(\frac{4c\sigma {\sigma }_{1}t\tau }{{\sigma }_{0}}+x+y\right)}{{\sigma }_{0}}\right)\right)}{q\left(\mathrm{sinh}\left(\frac{{\sigma }_{1}\tau \left(\frac{4c\sigma {\sigma }_{1}t\tau }{{\sigma }_{0}}+x+y\right)}{{\sigma }_{0}}\right)+\mathrm{cosh}\left(\frac{{\sigma }_{1}\tau \left(\frac{4c\sigma {\sigma }_{1}t\tau }{{\sigma }_{0}}+x+y\right)}{{\sigma }_{0}}\right)+d\right)}+{\sigma }_{0}^{2}+1.$$

**Type 4:** When $\rho \ne 0$ and $\tau \chi =0$,we obtained rational solutions. By putting constant values along with Eq. (32) and wave transformation Eq. (2) in the Eqs. (40) and (41) we get the rational solutions of Eqs. (33) and (34) such as

$${u}_{27}(x,y,t)=-\frac{{\delta }_{1}}{\sqrt{4{\delta }_{0}{\delta }_{2}-{\delta }_{1}^{2}}}+\frac{2{\delta }_{2}}{\sqrt{4{\delta }_{0}{\delta }_{2}-{\delta }_{1}^{2}}\left({d}_{1}+q\left(\frac{t\left(2{\delta }_{2}{\rho }^{2}+{\delta }_{1}^{2}-4{\delta }_{0}{\delta }_{2}-{\delta }_{2}\right)}{{\delta }_{2}\rho }+x+y\right)\right)},$$

$${v}_{27}(x,y,t)={\sigma }_{0}^{2}+1-\frac{2{\sigma }_{1}{\sigma }_{0}}{q\left(\frac{4c\sigma {\sigma }_{1}t\tau }{{\sigma }_{0}}+x+y\right)+{d}_{1}}+\frac{{\sigma }_{1}^{2}}{{\left(q\left(\frac{4c\sigma {\sigma }_{1}t\tau }{{\sigma }_{0}}+x+y\right)+{d}_{1}\right)}^{2}},$$

where ${d}_{1}$ is an arbitrary constant^44^.

## Graphical behaviors

**Figure 1:**
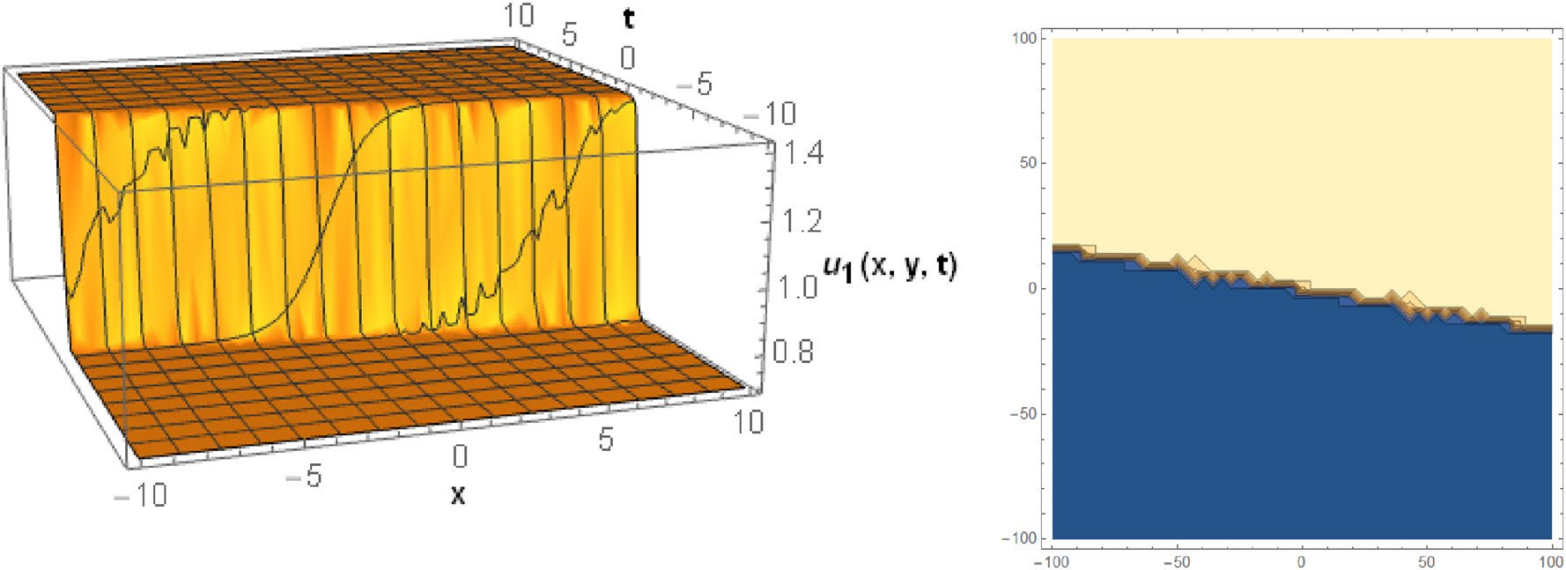
The plots ${u}_{1}$ for the values of constants ${\delta }_{0}=1.995,{\delta }_{1}=0.209,{\delta }_{2}=2.1,\rho =4.1,\tau =1.98,\chi =1.9$ and $y=1$.

**Figure 2:**
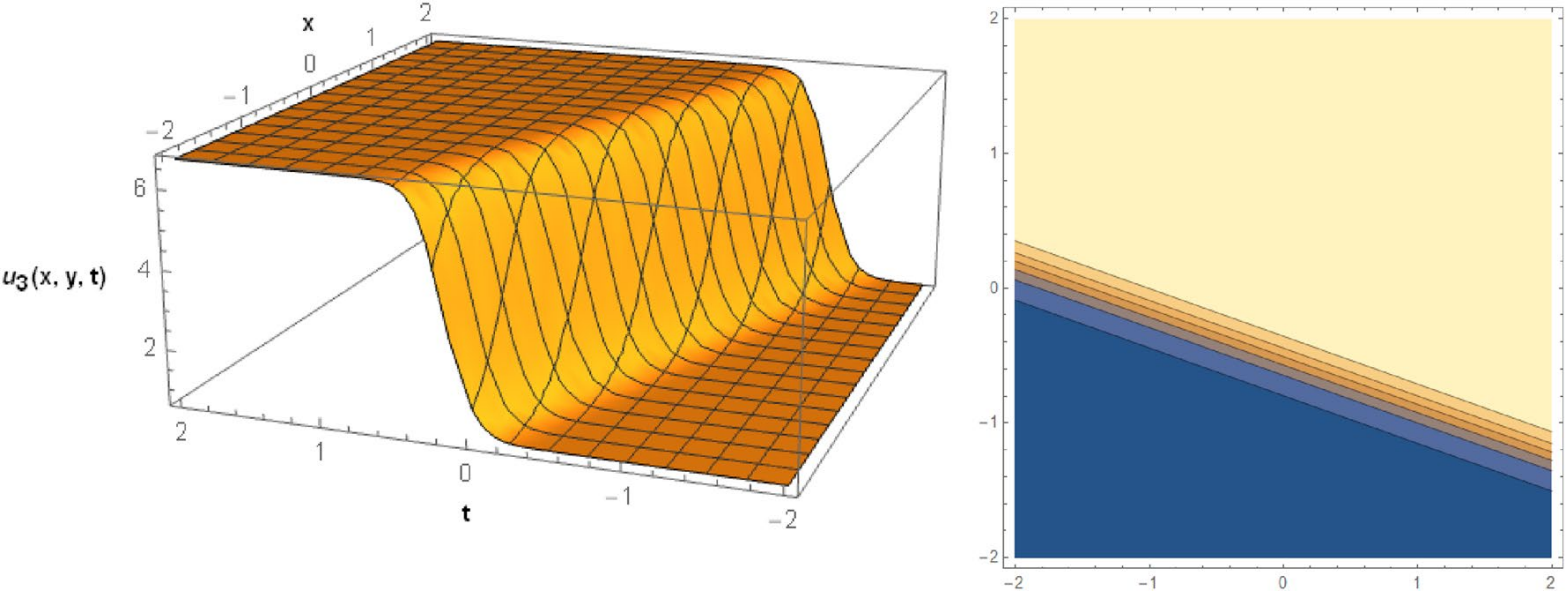
The 3D and 2D graphs for the values of ${u}_{3}$ for the values of parameters ${\delta }_{0}=1.909,{\delta }_{1}=0.00209,{\delta }_{2}=10.1,\rho =2.9,\tau =0.8,\chi =0.9,$ and $y=1.3$.

**Figure 3:**
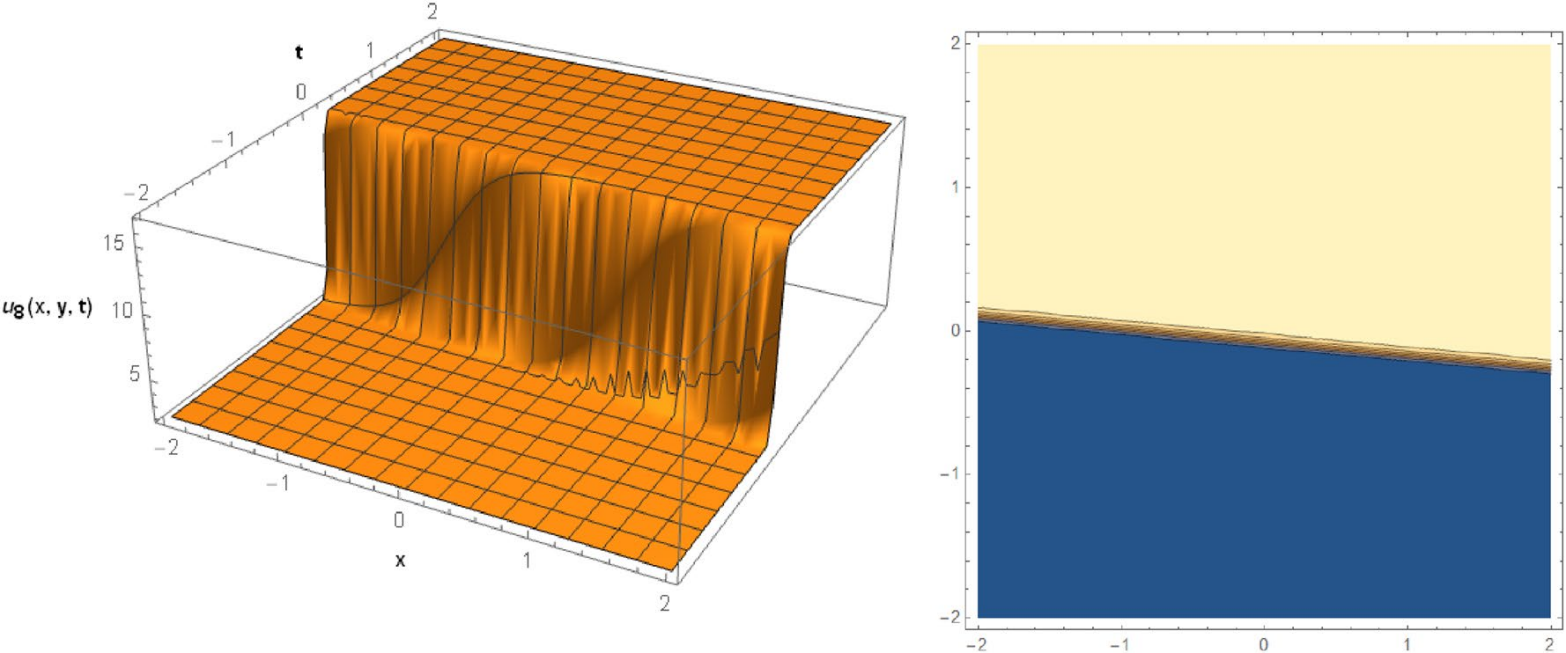
The plots ${u}_{8}$ for the values of constants ${\delta }_{0}=0.9,{\delta }_{1}=0.9,{\delta }_{2}=30.1,\rho =5.9,\tau =1.8,\chi =1.9,$ and $y=1.3$.

**Figure 4:**
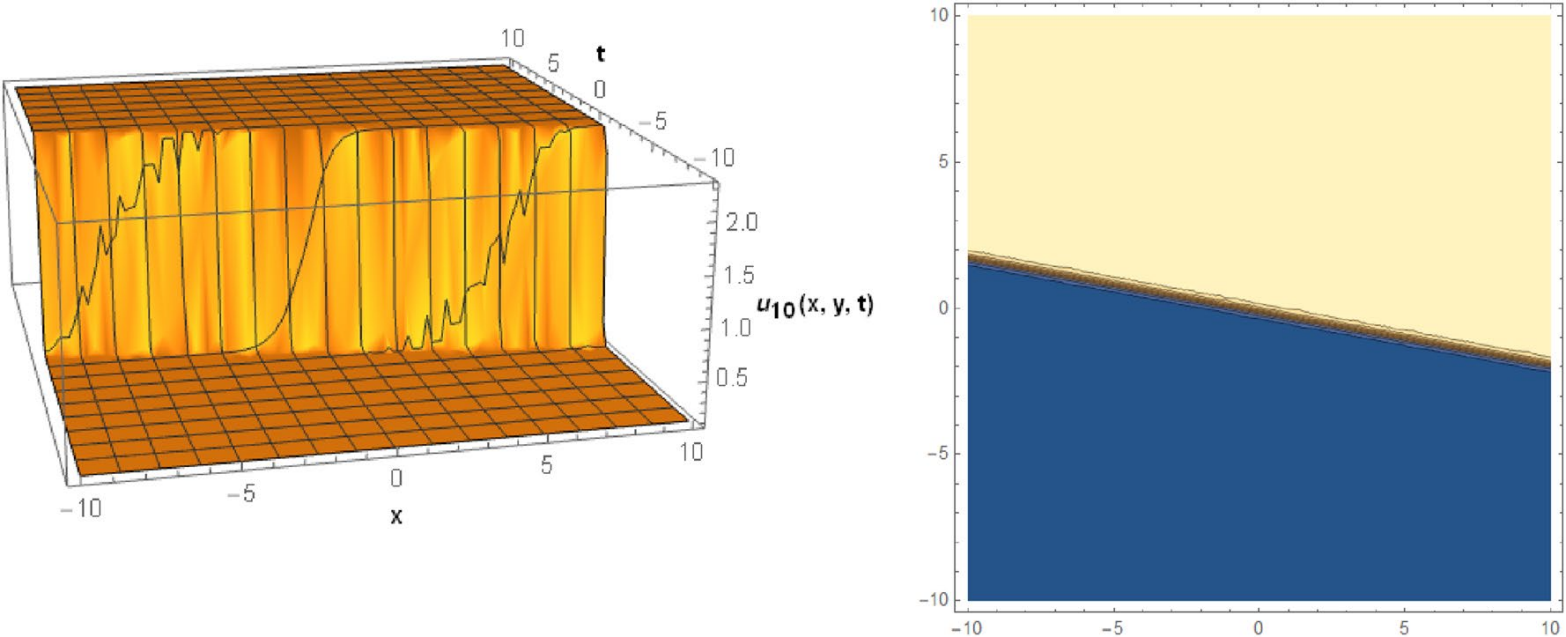
The plots ${u}_{10}$ for the values of constants ${\delta }_{0}=0.01,{\delta }_{1}=0.09,{\delta }_{2}=0.1,\rho =2.9,\tau =1.8,\chi =0.9,$ and $y=1$.

**Figure 5:**
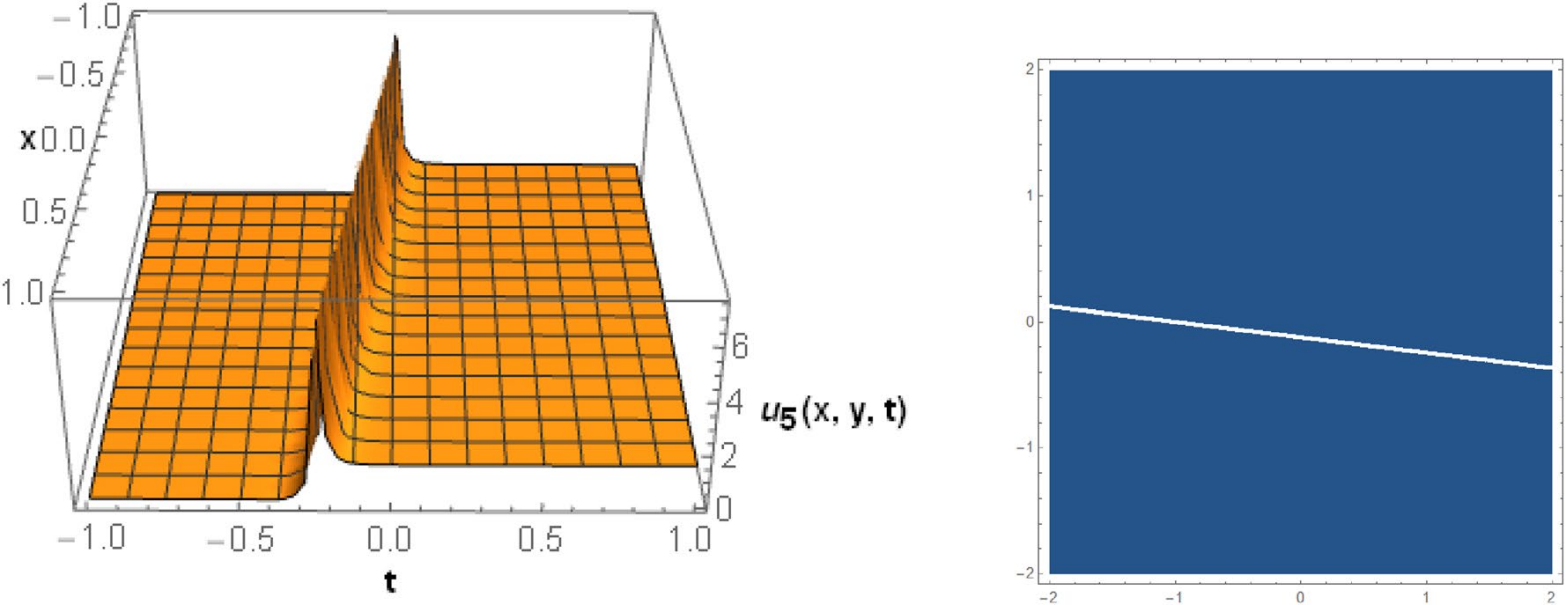
The plots ${u}_{5}$ for the values of constants ${\delta }_{0}=1.909,{\delta }_{1}=0.9,{\delta }_{2}=1.1,\rho =4.9,\tau =1.8,\chi =1.9,$ and $y=1$.

**Figure 6:**
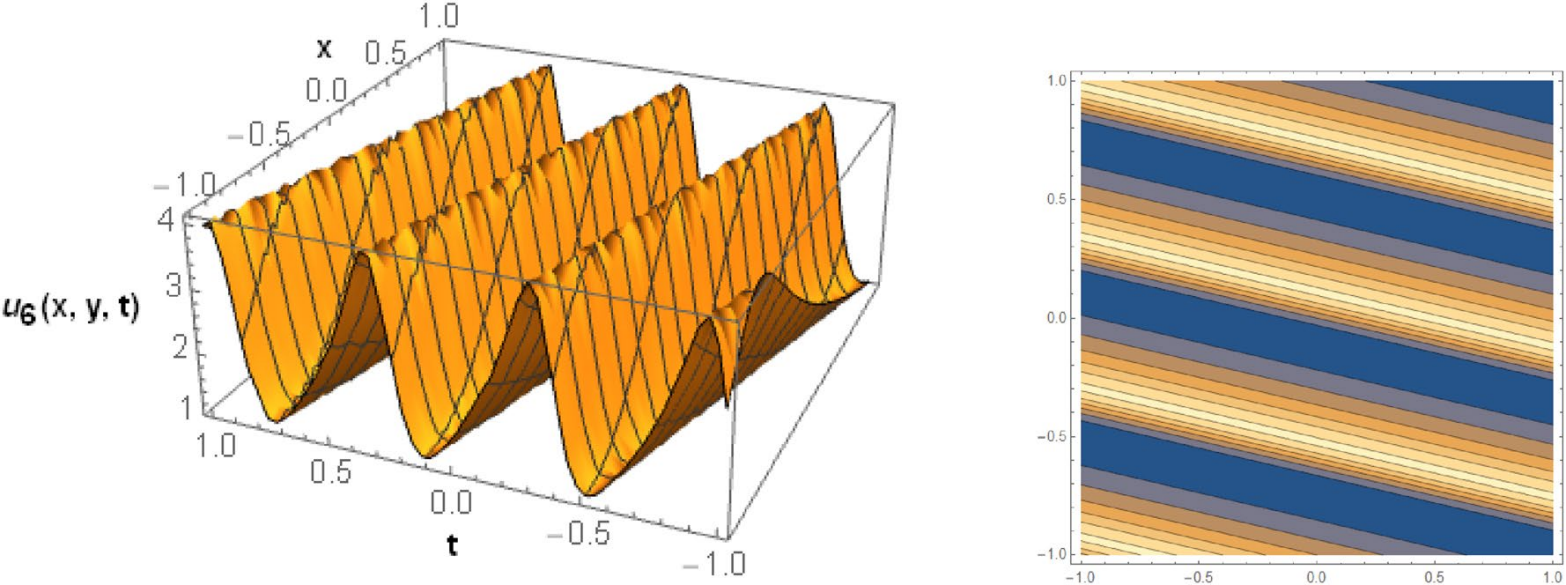
The plots ${u}_{6}$ for the values of constants ${\delta }_{0}=1,{\delta }_{1}=1.9,{\delta }_{2}=5.1,\rho =2.9,\tau =1.8,\chi =1.9,y=1$ and $H=G=2$.

**Figure 7:**
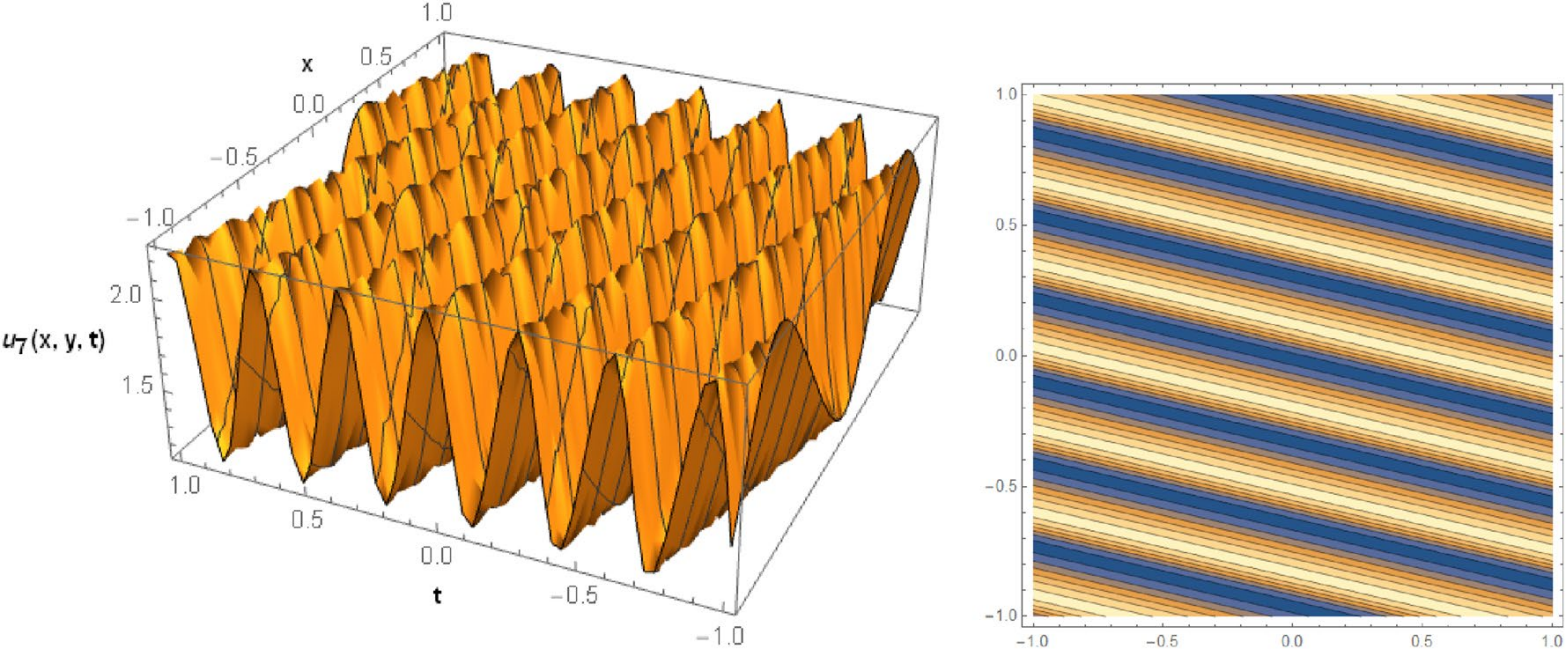
The plots ${u}_{7}$ for the values of constants ${\delta }_{0}=1,{\delta }_{1}=1.9,{\delta }_{2}=5.1,\rho =2.9,\tau =1.8,\chi =1.9,y=1,$ and $H=G=2$.

**Figure 8:**
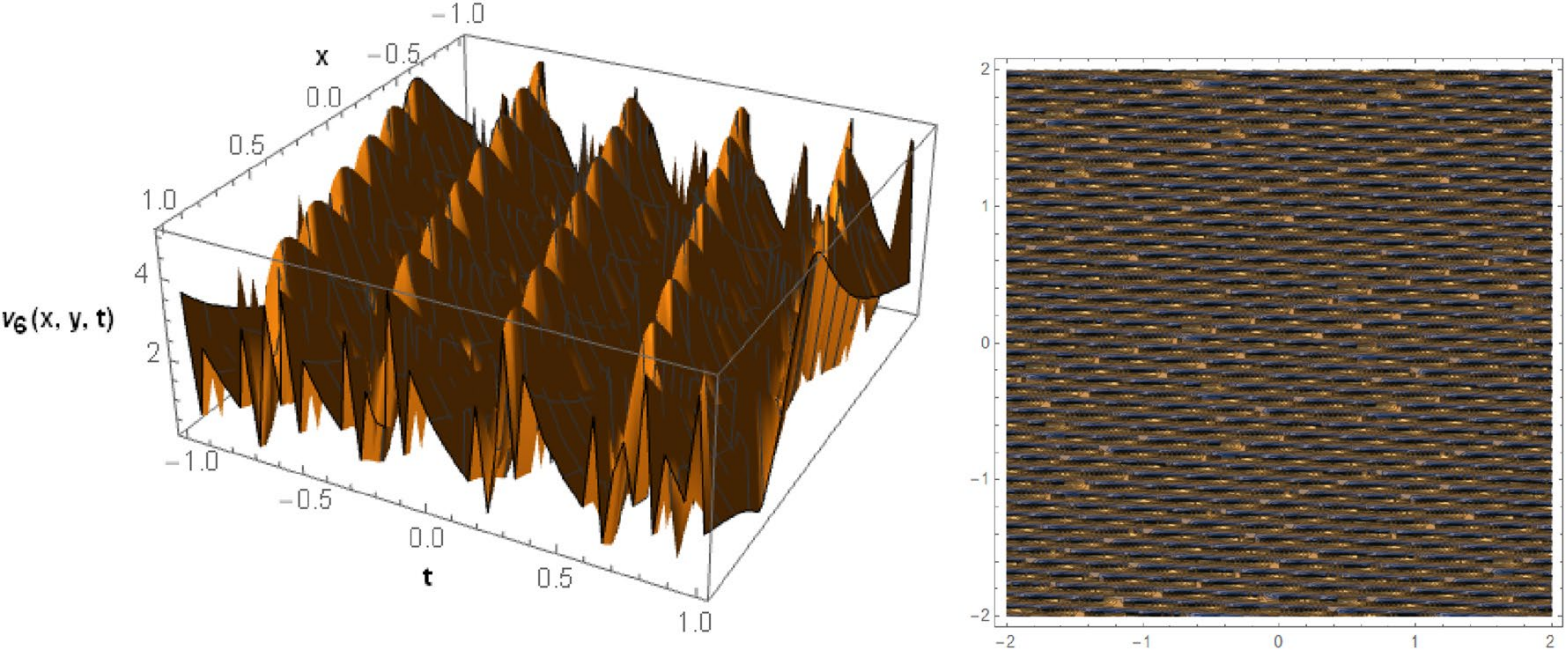
The plots ${v}_{6}$ for the values of constants $c=2.1,\rho =2.9,\sigma =1.9,{\sigma }_{0}=1.2,{\sigma }_{1}=0.9,\tau =1.8,\chi =1.9,y=1,H=G=3,$.

**Figure 9:**
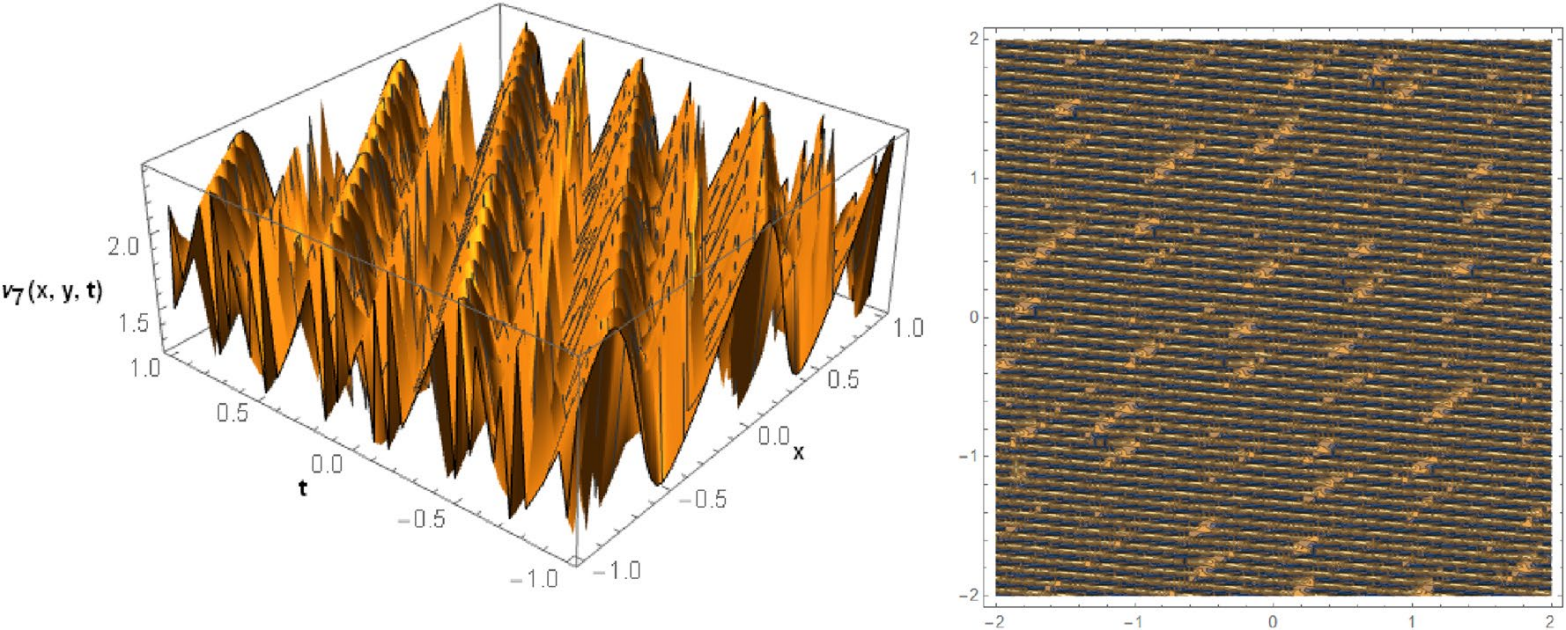
The plots ${v}_{7}$ for the values of constants $c=1.1,\rho =2.9,\sigma =1.9,{\sigma }_{0}=1.2,{\sigma }_{1}=0.9,\tau =1.8,\chi =1.9,y=1,$ and $H=G=3$.

**Figure 10:**
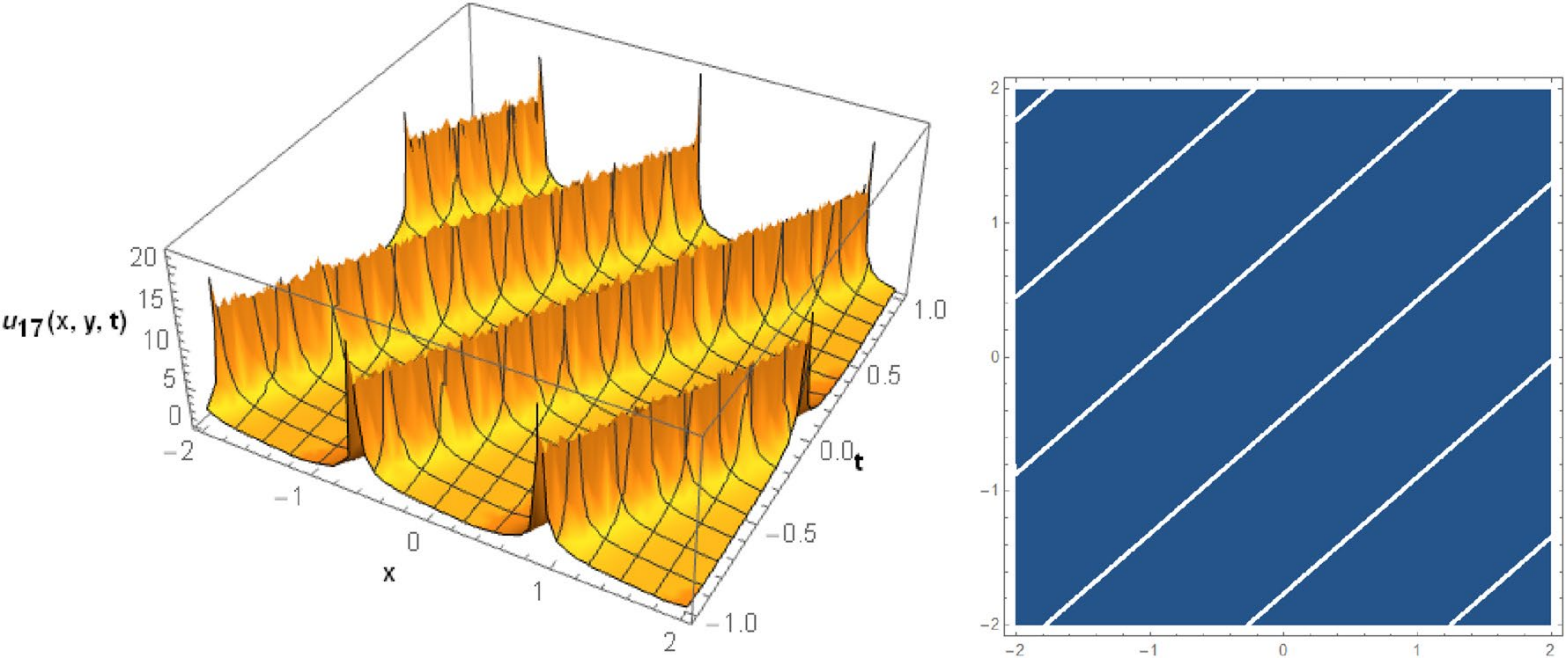
The plots ${u}_{17}$ for the values of constants ${\delta }_{0}=2.1,{\delta }_{1}=0.009,{\delta }_{2}=1.1,\rho =1.9,\tau =1.8,\chi =2.9,$ and $y=1$.

**Figure 11:**
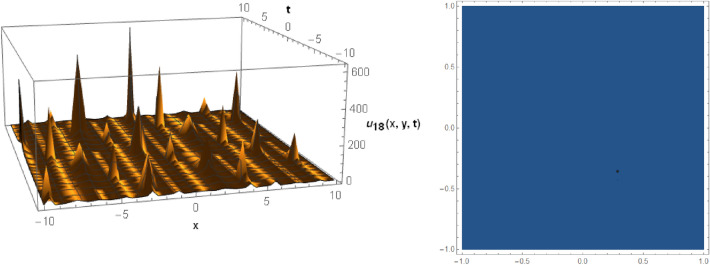
The plots ${u}_{18}$ for the values of constants ${\delta }_{0}=2.2,{\delta }_{1}=2.9,{\delta }_{2}=1.1,\rho =0.09,\tau =1.8,\chi =1.9,y=1,$ and $H=G=3$.

**Figure 12:**
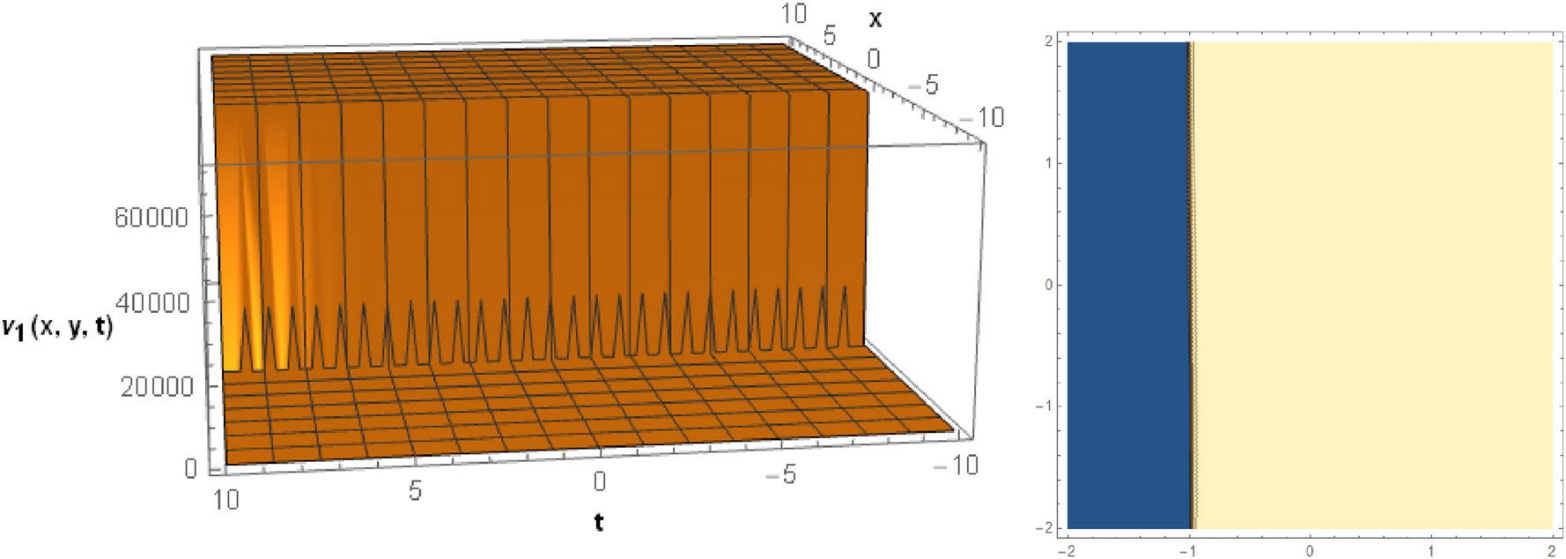
The plots ${v}_{1}$ for the values of constants $c=0.00001,\rho =3,\sigma =0.9,{\sigma }_{0}=0.01,{\sigma }_{1}=1.9,\tau =0.8,\chi =1.09,$ and $y=1$.

**Figure 13:**
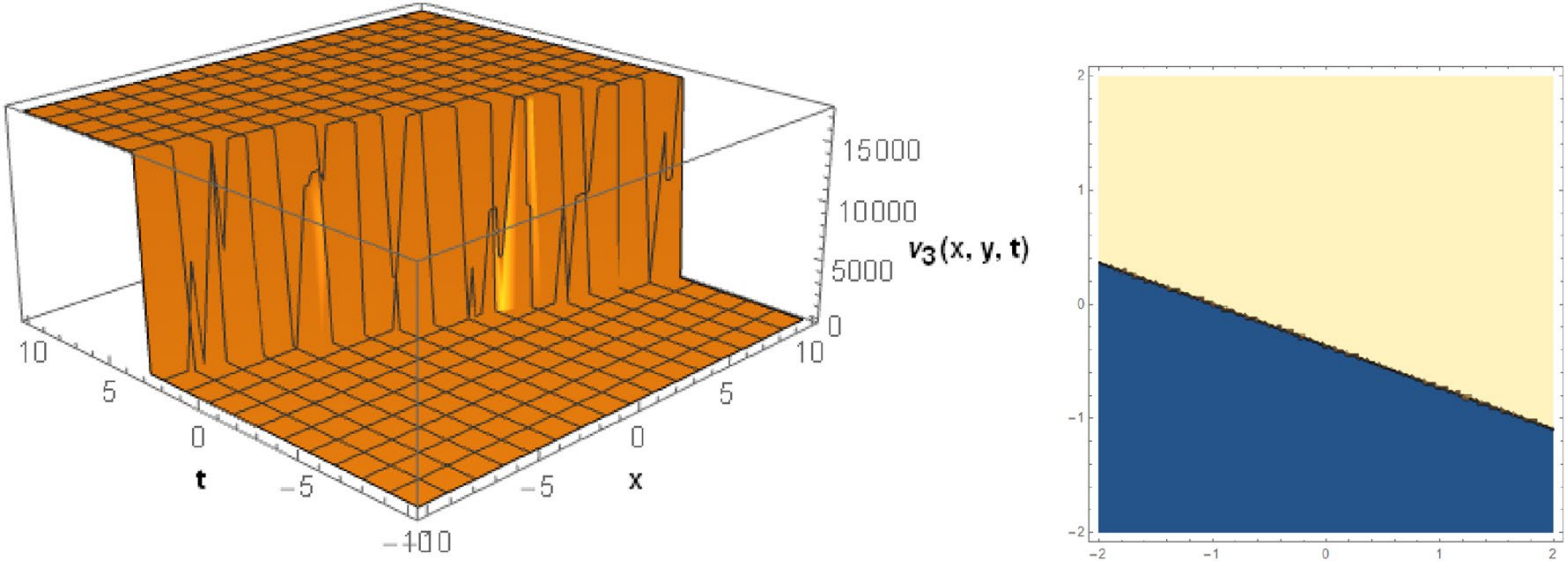
The plots ${v}_{3}$ for the values of constants $c=0.01,\rho =3,\sigma =0.9,{\sigma }_{0}=0.02,{\sigma }_{1}=1.9,\tau =0.8,\chi =1.09,$ and $y=1$.

**Figure 14:**
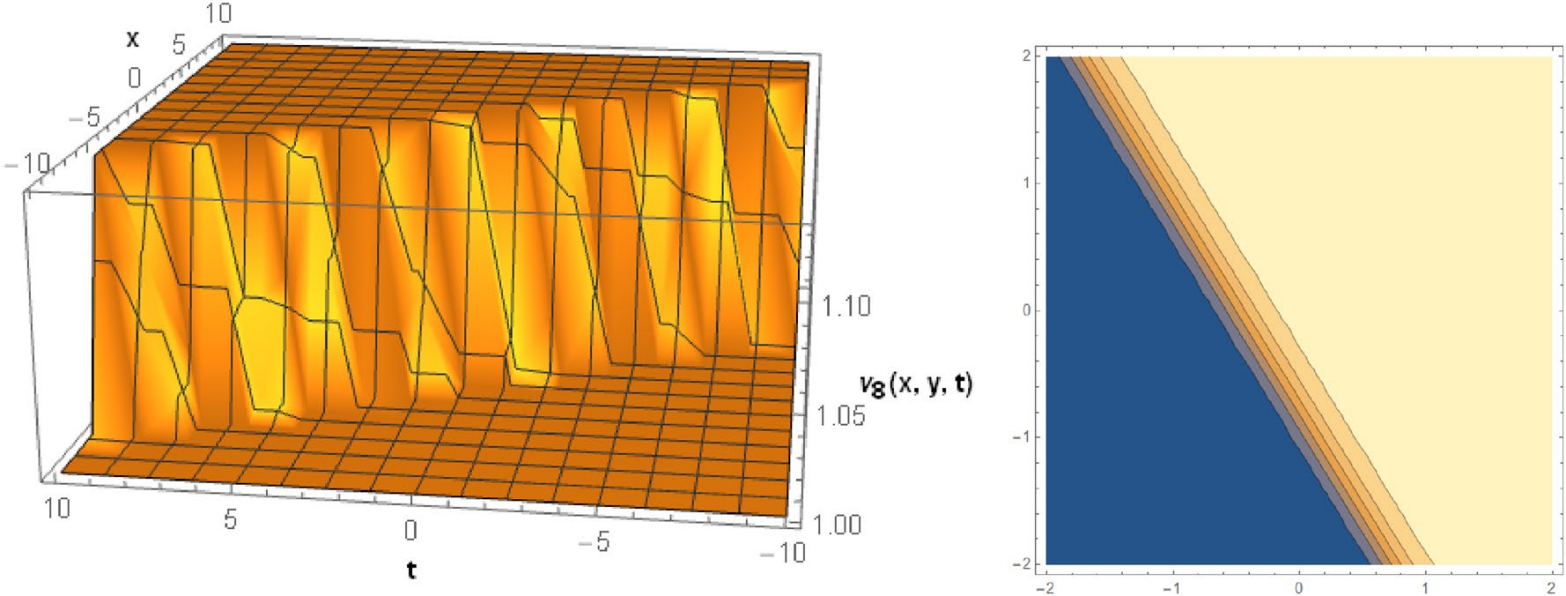
The plots ${v}_{8}$ for the values of constants $c=0.01,\rho =2.9,\sigma =1.9,{\sigma }_{0}=0.02,{\sigma }_{1}=0.09\tau =1.8,\chi =1.9,$ and $y=1$.

**Figure 15:**
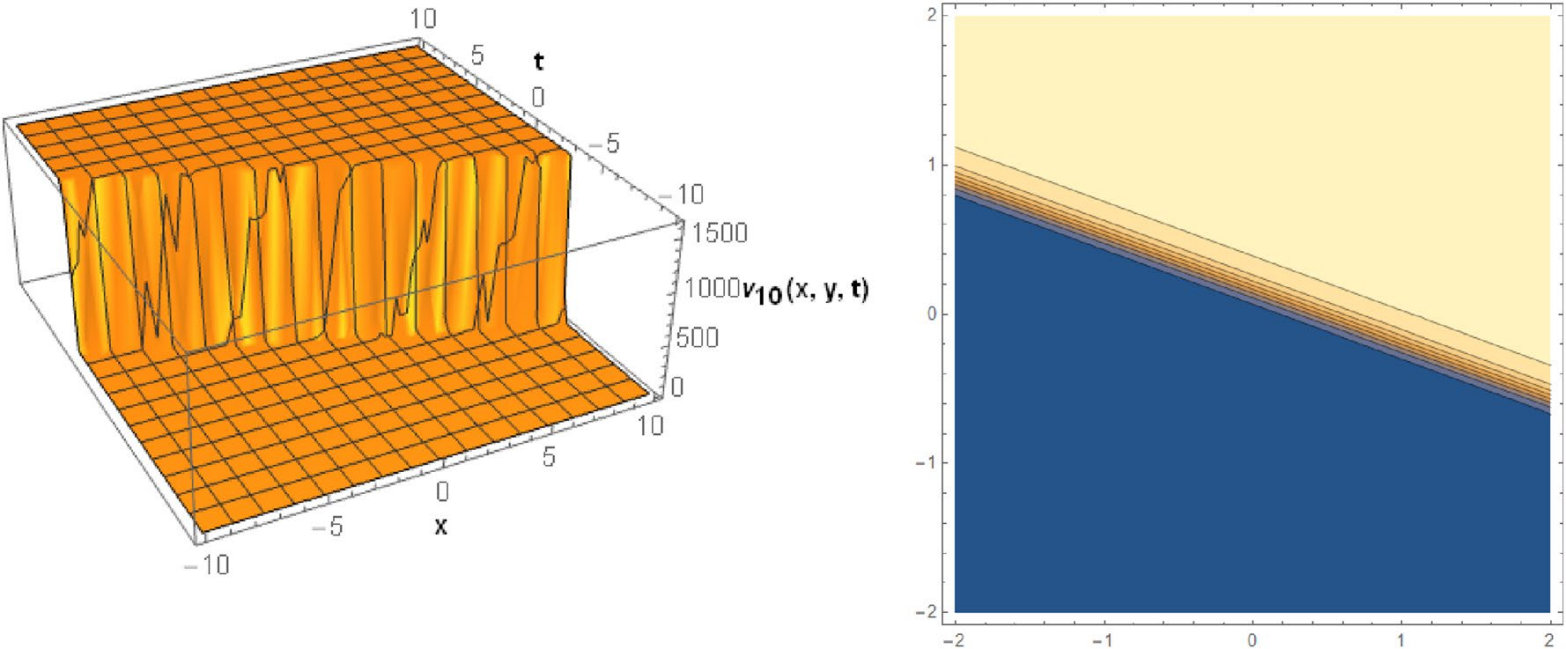
The plots ${v}_{10}$ for the values of constants $c=0.1,\rho =4.9,\sigma =1.9,{\sigma }_{0}=0.02,{\sigma }_{1}=0.9,\tau =0.08,\chi =0.9$ and $y=1$.

In this section, we discuss the graphical behavior of the solutions that are successfully obtained by using the GREM method for the reaction–diffusion Lengyel-Epstein system. In summary, the GREM technique is a useful tool to obtain the exact solitary wave solutions and control theory, especially for linear time-invariant systems. Its applicability to a wide variety of control issues is constrained by the linearity assumption, complexity, and limits mentioned earlier. When selecting whether to adopt GREM or look into other control approaches, engineers should take into account the unique characteristics of their systems and the issue at hand. Many physical significances are explained by sketching some three-dimensional diagrams and their corresponding contours for the acquired solutions. These figures give us a better understanding of the behavior of these solutions. The different solutions are plotted in 3D and their corresponding contour representations on the MATHEMATICA 11.1 for the different values of constants. These results are very helpful in the dynamic study of this chemical reaction model. The Figs. 1, 2, 3 and 4 show the kink type soliton behavior for the inhibitor chlorite using the range of space and temporal parameters [− 10,10] and [− 2,2] respectively. The Figs. 5, 6, 7, 8 and 9 show the solitary wave behaviors for the range of space and temporal parameters [− 1,1]. The Figs. 10 and 11 are the lump solitons using the range of space and temporal parameters [− 2,2] and [− 10,10] respectively. The Figs. 12, 13, 14 and 15 are plotted for the range of space and temporal parameters [− 10,10].

## Conclusions

In this study, we find the analytical wave solutions for the Lengyel-Epstein reaction–diffusion system. The reaction–diffusion The Lengyel-Epstein model represents the concentration of the inhibitor chlorite and the activator iodide, respectively. These concentrations of the inhibitor chlorite and the activator iodide are shown in the form of wave solutions. The generalized Riccati equation mapping method is used to find the analytical solutions. The Generalized Riccati Equation Mapping (GREM) method is a powerful analytical technique for solving a wide range of differential equations, particularly nonlinear ones. The shock, complicated solitary-shock, shock singular, and periodic-singular wave solutions are seen for both single and mixed wave solutions. The derivation also leads to reasonable solutions. Solitary waves in the Lengyel-Epstein system can spread at different rates. The balance between a system’s diffusive and reactive effects typically controls how quickly a single wave travels. Depending on the variables and the kinetics of the response, solitary waves can move at a variety of speeds. Many physical significances are explained by sketching some three-dimensional diagrams and their corresponding contours for the acquired solutions. These figures give us a better understanding of the behavior of these solutions. These results are very helpful in the dynamic study of this chemical reaction model.

## Data Availability

The datasets used and/or analysed during the current study available from the corresponding author on reasonable request.
